# Interior Microstates and Black Hole Entropy

**DOI:** 10.3390/e28040408

**Published:** 2026-04-03

**Authors:** Martin Sasieta

**Affiliations:** Leinweber Institute for Theoretical Physics, University of California, Berkeley, CA 94720, USA; msasieta@berkeley.edu

**Keywords:** black holes, black hole entropy, gravitational path integral, holography, ads/cft, quantum gravity, black hole microstates, string theory

## Abstract

Semiclassical gravity admits a vast set of candidate black hole interior states, raising the question of which of these correspond to independent quantum microstates that account for black hole entropy. In this review, we survey several explicit constructions of black hole interior microstates in AdS_2_ holography and AdS/CFT and assess whether they furnish bases of the black hole Hilbert space. We further highlight the settings in which non-perturbative effects in the gravitational path integral, captured by spacetime wormholes, resolve the resulting overcounting and reproduce the black hole entropy from state counting.

## 1. Introduction

The problem of identifying black hole microstates is often framed as a deep mystery, given that in General Relativity, only a few macroscopic parameters (mass *M*, charge *Q*, and angular momentum *J*) determine the exterior spacetime of a black hole in equilibrium. Meanwhile, it is said that the underlying microstates must account for the large Bekenstein–Hawking entropy [1,2]

(1) $$S_{\mathrm{BH}}=\frac{A}{4G_{N}}\;.$$

**Figure 1:**
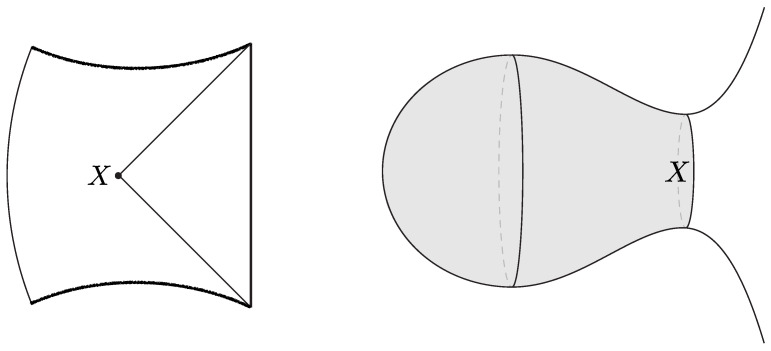
(**Left)** Penrose diagram of a spherical time-symmetric bag of gold spacetime in AdS for a black hole in equilibrium. (**Right**) Geometry of the time-symmetric slice, containing the horizon *X*.

However, the real puzzle is the opposite: there are far too many candidate states already present in the low-energy effective description of the black hole. This overabundance originates in the black hole interior, which semiclassically can accommodate an arbitrarily large entropy. (There is a different infinite-entropy puzzle associated with short-distance horizon physics: quantum fields on a black hole background exhibit a continuous energy spectrum. The discreteness is expected to be resolved by the short-distance structure of the bulk gravitational theory, unlike the situation relevant to this review.) At the classical level, this freedom is reflected in Wheeler’s bag of gold spacetimes [3], interior geometries behind the apparent horizon that cap off smoothly (see Figure 1). Because the interior volume can be arbitrarily large, the space of such configurations is correspondingly infinite. Treating these as valid states that approximate true microstates would then produce a vastly overcomplete set of states far exceeding the $e^{S_{\mathrm{BH}}}$ linearly independent states allowed by (1).

A natural framework for addressing this overcompleteness, without leaving the low-energy effective theory, is the gravitational path integral (GPI). (Historically, GPI derivations of black hole entropy originate in the work of Gibbons and Hawking [4]. Unlike the derivation reviewed here, the Gibbons–Hawking derivation does not involve an explicit state counting, and its quantum statistical interpretation is not manifest.) This perspective has come into focus recently, largely through an improved understanding of the role of spacetime wormholes in the GPI. The prevailing view is that the GPI, including wormholes, provides access to non-perturbative yet suitably coarse-grained information about the microscopic holographic system within its large-*N* or semiclassical expansion. This information is coarse-grained through “diagonal” or “phase-correlated” approximations to the chaotic properties of the holographic system.

In the present context, spacetime wormhole contributions to the GPI detect non-perturbative overlap moments among different black hole interior microstates. This reveals that many semiclassically distinct interior configurations fail to define orthogonal vectors in the holographic Hilbert space. As a result, the Hilbert space dimension spanned by infinite families of microstates is drastically reduced. When the state counting is properly performed using the GPI, all such families are found to span the same Hilbert space, of dimension $e^{S_{\mathrm{BH}}}$. In this way, black hole entropy (1) is reproduced via state counting.

The scope of this review is twofold. First, a variety of concrete constructions of black hole interior microstates have been developed in AdS_2_ holography and AdS/CFT, each realizing a distinct class of black hole interior. Section 2 and Section 3 survey several representative families of such microstates, ranging from constructions that admit well-controlled Euclidean preparations to others that are more speculative or remain incomplete. We assess the extent to which each family can furnish a basis for the black hole Hilbert space. Secondly, in many of these constructions, it is now understood how a proper state counting using the GPI yields a finite entropy consistent with (1). In Section 4, we review the mechanism underlying this state counting and the concrete Euclidean wormholes responsible for it.

## 2. A Plethora of Black Hole Microstates

In this section, we classify known constructions of black hole microstates in holography. We focus on pure states of a single black hole. Given the Hamiltonian *H* of the black hole, all the microstates we will review admit a representation of the form

(2) $$|\Psi \rangle =\frac{e^{-\tau H}|W\rangle }{\sqrt{\langle W|e^{-2\tau H}|W\rangle }}\;,$$

where

(3) $$|W\rangle =\sum_{i}w_{i}|E_{i}\rangle \;,$$

is a (not necessarily normalized) pure state whose definition is independent of *H*. Here, $|E_{i}\rangle$ is an eigenstate of *H* with energy $E_{i}$. The complex coefficients $w_{i}$ and the real parameter τ define the precise microstate.

States of the form (2) are taken to define black hole microstates when they represent thermal pure states (TPSs), meaning that they appear thermalized: expectation values of sufficiently “simple” probes ϕ agree, in the large-*N* limit of the holographic system, with those computed in a thermal ensemble (for black holes in AdS, the large-*N* limit replaces the usual thermodynamic infinite volume limit, providing a limit in which the density of holographic degrees of freedom per thermal-scale area of the horizon diverges),

(4) $$\;\;\langle \Psi |\phi |\Psi \rangle ∼\mathrm{Tr}\left(\rho_{\beta }\phi \right)\;,\;\left(N\to \infty \right)\;.$$

Here, $\rho_{\beta }=\exp\left(-\beta H\right)/Z\left(\beta \right)$ is the Gibbs state at inverse temperature β and Z(β)=Tr(exp(−βH)) is the canonical partition function. We denote this equivalence to the thermal ensemble (TPS condition) as

(5) $$|\Psi \rangle ∼\rho_{\beta }\;.$$

The value of β in (5) is determined by the energy of the microstate $\langle \Psi |H|\Psi \rangle =\mathrm{Tr}\left(\rho_{\beta }H\right)$.

The definition of a TPS requires a more precise notion of a “simple” operator. Here, it is customary to restrict to operators describing $O(N^{0})$ quantum fields in the black hole exterior, such as those for Hawking modes of sufficiently short proper wavelength and small angular momentum (of energy well below the cutoff scale), and to probing timescales that do not scale with *N*. In AdS/CFT, this is commonly referred to as the large-*N* algebra of single-trace CFT operators (see [5] and references therein). Physically, this corresponds to simple experiments in the exterior and in the Hawking radiation, which would be accessible in practice.

A straightforward example of a microstate is a random, or “typical” TPS, where |*W*〉 is drawn randomly from the uniform Haar measure and τ=β/2. (In [6], typical TPSs were dubbed “thermal pure quantum states”.) In this case, the condition (5) is satisfied with extremely high probability by an individual state |Ψ〉. This is sometimes interpreted as an indication that a typical TPS of black holes will have the same exterior geometry and thermal properties as the equilibrium black hole (at least as measured by the large-*N* algebra). However, what the black hole interior looks like for truly typical TPSs, and whether the very notion of an interior exists at all, remains an open problem.

In Table 1, we summarize known constructions of black hole interior states that form TPSs and indicate whether the corresponding families of microstates are overcomplete.

### 2.1. EoW Particle States

Near-extremal black holes develop a nearly AdS_2_ throat with compact transverse dimensions whose low-energy gravitational dynamics are universally governed by Jackiw–Teitelboim (JT) dilaton gravity (see [41] for a comprehensive review). In AdS units ($\ell_{\mathrm{AdS}}=1$), the spacetime geometry of the throat in Poincaré coordinates is

(6) $$\;ds^{2}=\frac{-dt^{2}+dz^{2}}{z^{2}}\;.$$

The gravitational degree of freedom is the cutoff boundary close to z=0, and its trajectory is parametrized by $z\left(u\right)=\epsilon t^{\prime }\left(u\right)$ and t=t(u) as ϵ→0, with Schwarzian action $I_{\mathrm{Sch}}=-\phi_{b}\int du\left\{t\left(u\right),u\right\}$ where $\phi_{b}$ is the boundary value of the dilaton [42]. The black hole solutions take the form

(7) $$t\left(u\right)=\tanh\left(\frac{\pi u}{\beta }\right)\;,$$

where β is the inverse temperature of the black hole. This solution describes a constant radius trajectory in Rindler–AdS coordinates $ds^{2}=-f\left(r\right)dt_{R}^{2}+\;dr^{2}/f(r)$ with $f\left(r\right)=r^{2}-r_{h}^{2}$ and the horizon radius $r_{h}=2\pi /\beta$. The dilaton has a linear radial profile $\Phi \left(r\right)=\phi_{b}r$ and is defined on the remaining regions of the Poincaré patch by continuation beyond $r=r_{h}$.

**Figure 2:**
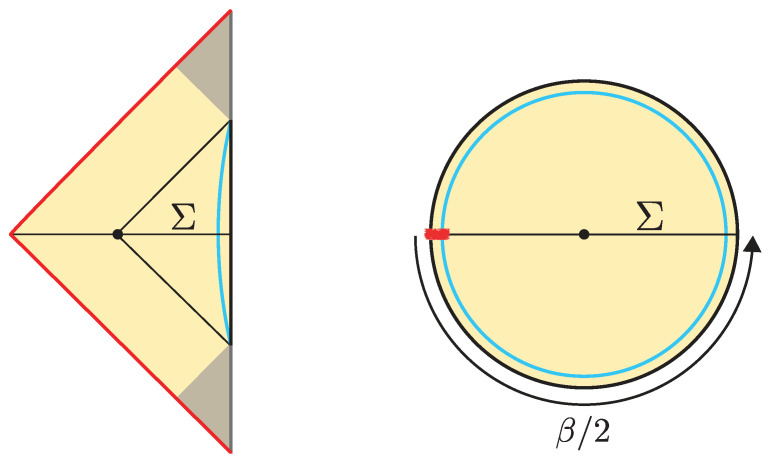
AdS_2_ (**left**) and hyperbolic disk (**right**) spacetimes for a microstate.

The black hole interior pure states are effectively modeled by coupling the JT theory to a very heavy end-of-the-world (EoW) particle [7]. (The EoW particle must be viewed as a simple effective model of the interior backreaction. In the putative bulk dual to the SYK model, the EoW particle should be replaced by a heavy source that effectively implements a (microstate-dependent) boundary condition for the O(N) bulk matter fields.) The EoW particle caps the spacetime at a large $z=z_{\infty }$ (the trajectory can be obtained from a $Z_{2}$-quotient of PETS states we review in Section 3.1). The resulting Lorentzian spacetime is represented in Figure 2. The cutoff boundary is shown in cyan, and the black hole exterior is delimited by the null rays that reach the boundary. These meet at the bifurcation point, which is the extremum of the dilaton. The horizontal black line Σ denotes the time-symmetric slice. This slice contains a large portion of the black hole interior, up to the EoW particle, and the dilaton grows toward the EoW particle in red. The shaded region lies behind the inner horizon of the black hole and is expected to be unstable under perturbations.

**Microstates.** The corresponding Kourkoulou–Maldacena (KM) microstates are defined in the Hilbert space of the SYK model, which consists of *N* Majorana fermions $\psi_{1},\ldots ,\psi_{N}$ with anticommutation relations $\left\{\psi_{i},\psi_{j}\right\}=2\delta_{ij}$ (see [43,44]). The Majoranas can be paired into N/2 Dirac fermions, with creation and annihilation operators $c_{i}^{†}=\left(\psi_{i}+i\psi_{N-i}\right)/\sqrt{2}$ and $c_{i}=\left(\psi_{i}-i\psi_{N-i}\right)/\sqrt{2}$, respectively, for i=1,…,N/2 (we assume even *N*). A natural basis of the SYK Hilbert space is provided by all product states $\left|P\rangle =\right|s_{1}s_{2}\ldots s_{N/2}\rangle$ of the Dirac fermions, common eigenstates of the occupation operators $S_{i}=2c_{i}^{†}c_{i}-1$, with eigenvalues

(8) $$S_{i}|P\rangle =s_{i}|P\rangle ,\;s_{i}\in \left\{-1,1\right\}\;.$$

These states are simple in the Dirac fermion basis but correspond to highly excited superpositions of energy eigenstates of the SYK Hamiltonian *H* of O(JN) energy above the ground state, where

(9) H=iq/2∑1≤i1<…<iq≤NJi1…iqψi1…ψiq ,withJi1…iq2¯=J2N2q2Nq .

Recall that the SYK couplings $J_{i_{1}\ldots i_{q}}$ are drawn independently from a Gaussian random distribution with zero mean and variance defined in (9).

The KM microstate is defined by the Euclidean time evolution (“cooling”) of a product state with the SYK Hamiltonian,

(10) $$|\Psi \rangle =\frac{e^{-\frac{\beta }{2}H}|P\rangle }{\sqrt{\langle P|e^{-\beta H}|P\rangle }}\;,$$

with Jβ≫1, so that the resulting microstate lies deep in the low-temperature regime, admitting a JT gravitational dual. The resulting family of KM microstates (for the $2^{N/2}$ different choices of |*P*〉) is highly overcomplete in any low-energy microcanonical window of the SYK spectrum.

The correspondence with the semiclassical interior geometry relies on the fact that at leading orders in *N* (up to order $1/N^{q-1}$ with respect to the leading answer), SYK correlation functions are self-averaging over couplings, and the model develops a symmetry $\psi_{i}\to M_{i}^{j}\psi_{j}$ for M∈O(N). Within this group, there is a “flip subgroup” of transformations that transform $\psi_{i}\to -\psi_{i}$ for i=N/2,…,N. The set of all product states of the form $|P\rangle =|s_{1}\ldots s_{N/2}\rangle$ forms an orbit under such a subgroup. Therefore, the flip-invariant operators, such as the Majorana bilinears $\phi =i\psi_{1}\left(\tau_{1}\right)\psi_{1}\left(\tau_{2}\right)$, have identical expectation values for any microstate of the form (10). These values are exactly thermal because of the orthonormality of the |*P*〉 basis. These thermal two-point functions fix the background geometry to be that of the Euclidean disk. Additionally, other non flip-invariant operators signal that |*P*〉 inserts a localized defect in Euclidean time. This defect (in red) can be interpreted as a boundary condition for the fermion fields via a (microstate-dependent) heavy source. An effective model for its backreaction in spacetime is a very heavy EoW particle. Under analytic continuation along the time-symmetric slice Σ, this yields the Lorentzian spacetime described above. (A noteworthy feature of the KM construction is that Lorentzian time-evolving the state with a suitably perturbed Hamiltonian, involving a microstate-dependent bilocal term, can make the black hole interior causally accessible [7].)

**Generalizations and relation to other constructions.** States similar to KM microstates have been studied in spin chains under the name of “minimally entangled typical thermal states” (METTS) [45,46,47]. This term can be misleading in the present context. Throughout this review, we use the term TPS rather than “typical thermal state” and reserve typical to mean random. Moreover, the KM states are not expected to exhibit low entanglement between subsystems of fermions. Unlike the Lorentzian evolution, which is constrained in how entanglement can grow, the Euclidean evolution used to prepare the microstates generates large amounts of entanglement. (For example, see Appendix C of [19].)

The closest candidates for higher-dimensional versions of KM states may be the EoW brane states we review in Section 2.3. In fact, explicit constructions relating the two have been developed [48]. However, the higher-dimensional *B*-states are strongly constrained by conformal invariance (e.g., although they might admit a product structure in lattice systems at the critical point, they are homogeneous) and therefore constitute a restricted and highly atypical subclass of TPSs [47]. As a consequence, they are not expected to be overcomplete in the microcanonical window associated with the black hole, unlike KM states.

### 2.2. Dust Shell States

Codimension-one interfaces provide a simple way to treat the backreaction of matter in General Relativity. The interfaces glue together different spacetime regions according to the Israel–Darmois junction conditions [49]. Dust shells form a particularly basic class of such interfaces, modeling configurations of massive, uncharged particles. The corresponding energy–momentum tensor for a dust shell takes the form of a pressureless perfect fluid,

(11) $$T_{\mu \nu }=\sigma \;u_{\mu }u_{\nu }\;\delta \left(y\right)\;,$$

where σ is the mass density, $u^{\mu }$ is the proper velocity of the dust particles, and *y* is a proper Gaussian normal coordinate in the vicinity of the interface (with the shell’s worldvolume W located at y=0). Denoting $u^{\mu }={\left(\partial /\partial t_{s}\right)}^{\mu }$, for $t_{s}$ the proper time of the dust particles, the total rest mass of the shell is conserved along the proper time slicing of W and is given by

(12) $$m=\int_{S_{t}}da\;\sigma \;,$$

where $S_{t}$ denotes a spatial section of W at fixed $t_{s}=t$, and da is the induced volume element.

Much of the literature focuses on homogeneous and isotropic thin shells with worldvolume $W≅R_{t_{s}}\times Y$, where *Y* is a (d−1)-dimensional maximally symmetric space (in what follows $Y=S^{d-1}$). The intrinsic metric then takes the FRW form $ds_{W}^{2}=dt_{s}^{2}+R{\left(t_{s}\right)}^{2}\;ds_{Y}^{2}$, with $R(t_{s})$ representing the shell’s scale factor and $ds_{Y}^{2}$ representing the unit metric on *Y*.

**Single-shell states.** Among the known constructions of black hole interior states in AdS/CFT are configurations involving a single spherically symmetric dust shell of mass *m* [10,13,14,16]. The shell worldvolume W separates an interior region of empty AdS (−) from an exterior Schwarzschild–AdS geometry (+), with line elements ($\ell_{\mathrm{AdS}}=1$)

(13) $$ds_{\pm }^{2}=-f_{\pm }\left(r\right)dt_{\pm }^{2}+\frac{dr^{2}}{f_{\pm }\left(r\right)}+r^{2}d\Omega_{d-1}^{2}\;,\;\left\{\begin{array}{c} f_{+}\left(r\right)=1+r^{2}-\frac{16\pi GM}{\left(d-1\right)V_{\Omega }\;r^{d-2}}\;,\\ f_{-}\left(r\right)=1+r^{2}\;. \end{array}\right.$$

Here, $V_{\Omega }=\mathrm{Vol}\left(S^{d-1}\right)$ denotes the volume of the unit sphere and *M* is the ADM mass of the black hole. The two regions are glued along the shell’s trajectory $r\left(t_{\pm }\right)=R\left(t_{s}\left(t_{\pm }\right)\right)$, which obeys the equation of motion of a non-relativistic particle ${\dot{R}}^{2}+V_{s}\left(R\right)=0$ for the effective potential [13,50]

(14) $$V_{s}\left(R\right)=f_{+}\left(R\right)-{\left(\frac{M}{m}-\frac{4\pi Gm}{\left(d-1\right)V_{\Omega }R^{d-2}}\right)}^{2}\;.$$

**Figure 3:**
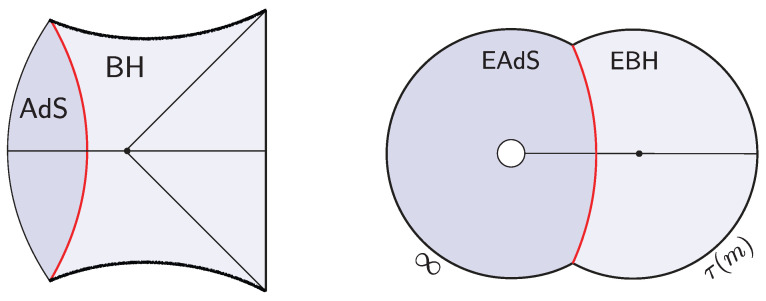
Lorentzian (**left**) and Euclidean (**right**) spacetimes for a single-shell microstate.

The shell’s trajectory $R(t_{s})$ starts at the past singularity $R(-t_{0}/2)=0$, reaches a maximal radius $R\left(0\right)=R_{*}$ (for which $V_{s}\left(R_{*}\right)=0$), and finally collapses to the future singularity $R(t_{0}/2)=0$. Here, $t_{0}$ is the total proper time elapsed by the shell. The Penrose diagram of the resulting spacetime is shown in Figure 3.

The spacetime geometry is fully determined by two independent parameters: the ADM mass of the black hole *M* and the shell’s rest mass *m*. The former is fixed by the requirement that the exterior is a black hole geometry of a given mass, while the latter is a free parameter that controls the spatial extent of the black hole interior through the value of $R_{*}=R_{*}\left(m\right)$. Importantly, there is no upper bound on the shell’s rest mass *m* and, consequently, on the spatial volume of the black hole interior for single dust shell states.

**Microstates.** The corresponding CFT microstate is constructed by acting on the CFT vacuum |0〉 on the sphere with a dust shell operator O, which creates a spherical shell of rest mass *m* at a fixed radial position $r_{\infty }$ in the asymptotic AdS region (see Appendix A for details). This state is then evolved in Euclidean time by an amount τ using the CFT Hamiltonian *H*,

(15) $$|\Psi \rangle =\frac{e^{-\tau H}O|0\rangle }{\sqrt{\langle 0|O^{†}e^{-2\tau H}O|0\rangle }}\;.$$

The unnormalized microstate $e^{-\tau H}O|0\rangle$ is prepared by a Euclidean CFT path integral on a semi-infinite cylinder $\left(-\infty ,\tau \right]\times S^{d-1}$ with the Euclidean time interval (−∞,0] preparing the CFT vacuum |0〉 at $t_{E}=0$, the dust shell operator O inserted at $t_{E}=0$, and the interval (0,τ] providing the Euclidean evolution that cools the state down.

The correspondence with the semiclassical geometry follows from evaluating the norm of the microstate $\langle 0|O^{†}e^{-2\tau H}O|0\rangle$ at large-*N* via a saddle point of the corresponding bulk GPI. The dominant Euclidean saddle point geometry is shown in Figure 3. This saddle corresponds to a Euclidean black hole geometry glued to a Euclidean AdS region along the Euclidean worldvolume of the dust shell. The solution is obtained from (13) by analytic continuation, $t_{\pm }=it_{E,\pm }$. The resulting saddle prepares a pure state on the time-symmetric slice Σ, which serves as initial data for the Lorentzian spacetime described above. Importantly, the right Euclidean black hole includes its tip, so the shell begins its Lorentzian evolution behind the black hole horizon.

The cooling time τ sets the energy of the state and is fixed by requiring that it reproduces the ADM mass of the black hole, 〈Ψ|H|Ψ〉=M. Equivalently, the same condition follows from the requirement that the total Euclidean time periodicity of the black hole portion of the saddle equals the inverse temperature β(M). This yields the implicit equation

(16) $$\beta =2\tau +2\int_{R_{*}}^{\infty }\frac{dR}{f_{+}\left(r\right)}\sqrt{\frac{f_{+}\left(R\right)-V_{s}\left(R\right)}{V_{s}\left(R\right)}}\;,$$

which implies that β/4≤τ≤β/2. In the heavy shell limit, m/M→∞, with β finite, the trajectory of the shell “pinches off” in Euclidean time, $R_{*}\to \infty$, and one finds τ→β/2.

The microstate (15) forms a TPS of the black hole which satisfies (5) for sufficiently simple probes in the large-*N* limit. In the bulk, this is reflected in the fact that the exterior spacetime is identical to the Schwarzschild–AdS black hole solution. Although the state of the quantum fields is not exactly thermal, for sufficiently large shell masses, the deviations only affect very long-wavelength modes, which are not populated in the black hole atmosphere. The state also reaches a fully thermalized regime under relatively short Lorentzian time evolution.

Since the mass of the dust shell *m*, corresponding to the number of primary insertions in O, is unbounded above, the resulting family of dust shell microstates, for different shell masses, is highly overcomplete in any high-energy microcanonical Hilbert space of the CFT.

**Figure 4:**
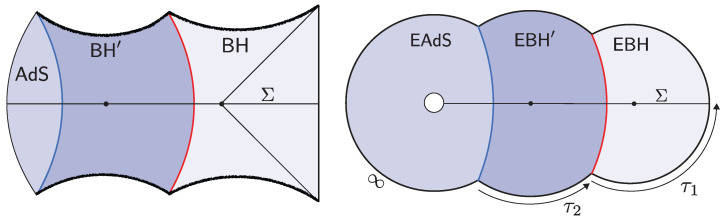
Lorentzian (**left**) and Euclidean (**right**) spacetimes for a two-shell state.

**Multi-shell states.** The construction above can be easily generalized to multi-shell states [13] (see Figure 4). The corresponding microstate for a *n*-shell state is

(17) $$|\Psi \rangle \;\propto \;e^{-\tau_{1}H}O_{1}e^{-\tau_{2}H}O_{2}\ldots e^{-\tau_{n}H}O_{n}|0\rangle .$$

The parameter $\tau_{1}\in \left(\frac{\beta }{4},\frac{\beta }{2}\right)$ is determined in terms of the rest $\tau_{i}$ for i=2,…,n, as well as of the shell’s masses $m_{j}$ for j=1,…,n, from the condition that the ADM mass is *M*. One important aspect of the construction to avoid subtleties in the Euclidean preparation is that the shells must be sufficiently distinct from one another. A way to accomplish this is to make the shells out of different primaries (or to make the mass differences $|m_{i}-m_{j}|$ sufficiently large) so that, in the large-*N* limit, different shells do not interact.

**Generalizations.** A number of generalizations of dust shell states have been explored in [16], showing that they provide a universal description of microstates for broader classes of black holes. One may also require the shells to carry a U(1) gauge charge or angular momentum. In the near-extremal charged black hole regime, the corresponding interior states can moreover be constructed directly in the higher-dimensional description, avoiding the near-horizon region.

Related pure states can also be constructed to describe the gravitational collapse of dust shells and the consequent formation of black holes [51] or processes in which a preexisting black hole absorbs a dust shell [52] (see [53,54] and Section 3.1 for related shockwave geometries). The corresponding microstates are out of equilibrium and, according to our definition (2), do not qualify as TPSs: simple operators in the exterior are expected to detect the presence of the shell. Although Lorentzian time evolution will eventually drive these states toward TPSs [53,54], they are not expected to account for any appreciable fraction of the final black hole entropy, reflecting the thermodynamic irreversibility of gravitational collapse into a black hole.

### 2.3. EoW Brane States

Another well-studied class of codimension-one objects in General Relativity consists of spatial EoW boundaries described by branes with a purely tensional equation of state,

(18) $$T_{\mu \nu }=-\varsigma \;h_{\mu \nu }\;\delta \left(y\right)\;.$$

Here, ς=(d−1)T/(8πG), with T>0 the constant brane tension (recall that we set $\ell_{\mathrm{AdS}}=1$), and $h_{\mu \nu }$ the metric induced on the brane’s worldvolume W, which is located at y=0.

EoW branes have been used to construct black hole interior states in AdS/CFT [10,17,18,19,20,21,22,23,24,25,26]. The spacetime geometry of these states coincides with the AdS–Schwarzschild (+) solution (13) with mass *M*, while spacetime simply terminates at W and does not extend beyond it. The intrinsic metric $h_{\mu \nu }$ takes the FRW form $ds_{W}^{2}=dt_{b}^{2}+R{\left(t_{b}\right)}^{2}\;ds_{Y}^{2}$, where $R(t_{b})$ is the brane’s scale factor, $t_{b}$ its proper time, and $Y=S^{d-1}$ in the present case. The stress-energy tensor (18) determines the brane’s trajectory ${\dot{R}}^{2}+V_{b}\left(R\right)=0$ for the effective potential

(19) $$V_{b}\left(R\right)=f_{+}\left(R\right)-{\left(TR\right)}^{2}\;.$$

**Figure 5:**
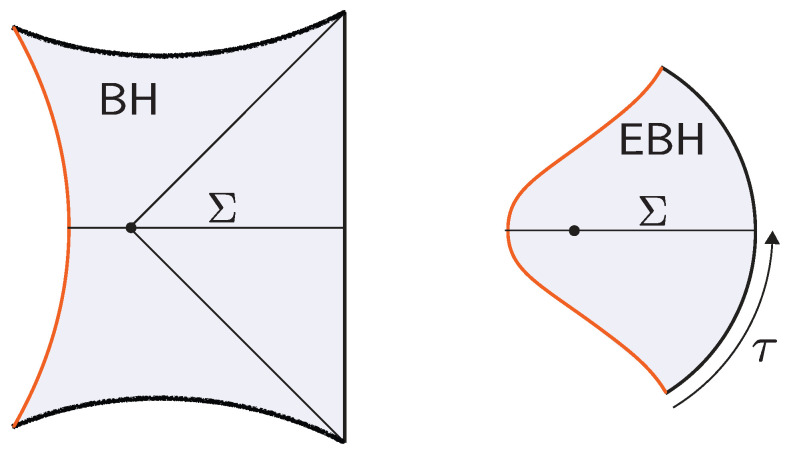
Lorentzian (**left**) and Euclidean (**right**) spacetimes for a *B*-state.

The brane begins at the past singularity, $R(-t_{0}/2)=0$, expands outward until it reaches a maximal radius $R\left(0\right)=R_{*}$ (satisfying $V_{b}\left(R_{*}\right)=0$), and then collapses back into the future singularity at $R(t_{0}/2)=0$. Such solutions exist provided the brane tension satisfies $T<T_{\mathrm{crit}}\left(M\right)$, where $T_{\mathrm{crit}}\left(M\right)\geq 1$ is a critical, mass-dependent value [19]. The corresponding Penrose diagram of the spacetime is shown in Figure 5.

**Microstates.** In boundary conformal field theory (BCFT), a central role is played by boundary (*B*-) states |*B*〉. These implement the possible conformal boundary conditions, which are infrared fixed points of the renormalization group flow of a CFT with a homogeneous boundary (see [55,56] and references therein). The relevant CFT microstates are constructed by evolving a *B*-state in Euclidean time,

(20) $$|\Psi \rangle =\frac{e^{-\tau H}|B\rangle }{\sqrt{\langle B|e^{-2\tau H}|B\rangle }}\;.$$

The unnormalized state $e^{-\tau H}|B\rangle$ is prepared by a Euclidean CFT path integral on a finite cylinder $\left[-\tau ,0\right]\times S^{d-1}$ with boundary conditions implementing |*B*〉 at Euclidean time $t_{E}=-\tau$.

In AdS/BCFT [57] (see also [58,59,60]), the holographic dual of |*B*〉 is realized by imposing an asymptotic boundary condition in the bulk for a purely tensional EoW brane. Bulk fields satisfy Neumann boundary conditions at the brane’s worldvolume W. (From a string-theoretic perspective, the EoW brane may be regarded as an effective description of supergravity backgrounds in which certain extra dimensions smoothly cap off [61,62].) The brane tension *T* is expected to control an appropriate notion of boundary entropy associated with the boundary state. This interpretation is most precise in CFT_2_, where *T* determines the boundary *g*-function [57].

The correspondence with the interior geometry then follows from the GPI evaluation of the norm $\langle B|e^{-2\tau H}|B\rangle$. For small τ, the dominant semiclassical saddle is a Euclidean black hole spacetime capped off by the Euclidean worldvolume of the EoW brane, as illustrated in Figure 5. The Euclidean time τ of the microstate is chosen such that 〈Ψ|H|Ψ〉=M or equivalently so that the total periodicity of the Euclidean black hole solution is fixed to β(M). This leads to the implicit relation

(21) $$\beta =2\tau +2\int_{R_{*}}^{\infty }\frac{dR}{f_{+}\left(r\right)}\sqrt{\frac{f_{+}\left(R\right)-V_{b}\left(R\right)}{V_{b}\left(R\right)}}\;,$$

which implies 0≤τ≤β/4. The identification with the microstate is only reliable for τ>0, or equivalently, using (21), when $T<T_{*}\left(M\right)$ for some tension $T_{*}\left(M\right)<1$ [19,63].

Unlike the previous examples, the microstates (20) can only form an undercomplete set of black hole microstates, accounting for at most a fraction of the black hole entropy [22]. The reason is that |*B*〉 is severely constrained by conformal invariance. In CFT_2_, *B*-states satisfy the gluing conditions $(L_{n}-{\bar{L}}_{-n})|B\rangle =0$ for the left- and right-moving Virasoro generators (and similarly for any extended chiral algebra), which impose infinitely many constraints and restrict |*B*〉 to the sector built from (h,h) representations. In holographic CFT_2_s, this implies that the number of independent such states scales at most like the square root of the full black hole Hilbert-space dimension; i.e., it carries at most half of the Cardy entropy. In higher dimensions, the corresponding fraction of the entropy can be estimated from the characteristic overlap between different EoW brane microstates [22].

**Braneworld cosmology.** Branes in AdS famously serve as models of large extra dimensions, in which a higher dimensional gravitational theory induces a local gravitational effective field theory on the brane’s worldvolume W [58,64]. For the EoW brane interior states, the braneworld metric is a crunching cosmological FRW spacetime with negative cosmological constant [65,66,67]. For gravity to localize on W (at least for some finite time before crunching), the brane must lie very far from the horizon of the black hole. For d>2, this can only be met parametrically in generalizations of the EoW brane states to charged black holes [21]. These braneworld theories become richer once other bulk fields are allowed in the black hole interior [26]. Analogous EoW brane interior states have been constructed for other types of black holes [23,25].

**Relation to other constructions.** The EoW boundaries are related to brane interfaces by $Z_{2}$-quotients across W. In the BCFT literature, this relation is commonly described as the “folding trick”, in which a conformal boundary is interpreted as a $Z_{2}$-quotient of a conformal defect. A natural generalization is to smear the defect, thereby realizing an extended interface between two CFTs. The holographic dual to this construction has been implemented for marginal deformations of holographic CFTs, where the bulk dual of the interface is a Janus solution with a nontrivial dilaton profile [68,69]. Janus interior states corresponding to entangled microstates of two holographic CFTs, of the class discussed in Section 3.1, were constructed in [70,71].

### 2.4. Caterpillar States

The previous constructions provide examples of often overcomplete families of black hole microstates, but the resulting TPSs are always atypical microstates. From the bulk perspective, the corresponding interiors are non-generic and largely empty. This raises the question of which classes of black hole interiors might instead give rise to more typical microstates.

**Figure 6:**
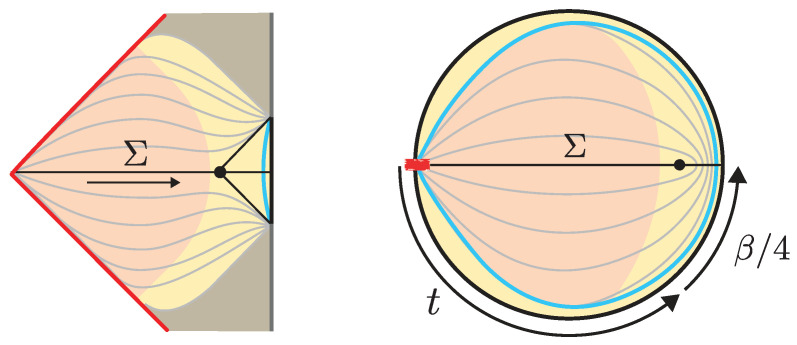
Lorentzian (**left**) and Eucidean (**right**) coarse-grained spacetimes for a pure state caterpillar. The deep interior (red region) develops an approximate spatial isometry ∂/∂x along constant dilaton slices (gray).

“Caterpillars” denote a class of black hole interior states characterized by long wormholes populated by inhomogeneous matter distributions [27,28]. (The term “caterpillar” was coined in [72].) Such interiors are expected to provide a quantitative approximation to typical microstates. The near-extremal black hole provides a particularly tractable example in which this behavior can be analyzed explicitly. Upon coarse graining over the matter inhomogeneity scale of the caterpillar, the resulting interior geometry is well described by an AdS_2_ metric in FRW slicing

(22) $$ds^{2}=\frac{-dt_{c}^{2}+dx^{2}}{\cosh^{2}\left(t_{c}\right)}\;,\;\mathrm{with}\;0\leq x+x_{\infty }≲t\;.$$

where $t_{c}$ is the conformal time. In particular, the interior spacetime develops a spatial isometry generated by ∂/∂x. The profile of the dilaton respects this isometry and $\Phi \left(t_{c}\right)∼-\left|t_{c}\right|$ for large $|t_{c}|$. (From the higher-dimensional near-extremal black hole perspective, the interior spacetime is an approximately homogeneous but anisotropic Big bang/Big crunch cosmology with cylindrical slices (with two different scale factors: one for the longitudinal direction and one for the spheres).) Recall that this solution is possible because the interior is filled with a matter distribution with a non-vanishing $T_{\mu \nu }$ (the metric is still AdS_2_ because of the minimal coupling of matter). At $x=-x_{\infty }+O\left(t\right)$, the interior is glued to the exterior Rindler wedge, where the boundary particle follows the trajectory (7). Here, *t* is a parameter that characterizes the length of the interior for a caterpillar. The interior terminates at some $x=-x_{\infty }$ where an EoW particle is inserted. The resulting spacetime is illustrated in Figure 6.

While the coarse-grained geometry is captured by this homogeneous description, at scales below the inhomogeneity scale, a caterpillar exhibits a locally irregular structure.

**Microstates.** In SYK, caterpillar microstates are constructed by applying an operator to an initial product state, which is followed by Euclidean time evolution of the resulting state

(23) $$|\Psi_{t}\rangle =\frac{e^{-\frac{\beta }{4}H}W\left(t\right)|P\rangle }{\sqrt{\langle P|W{\left(t\right)}^{†}e^{-\frac{\beta }{2}H}W\left(t\right)|P\rangle }}\;.$$

Here, *t* parametrizes a fictitious “circuit time” as the operator W(t) is generated by time-dependent Hamiltonian evolution

(24) $$\begin{array}{c} W\left(t\right)=T\left\{\exp\left(-i\int_{0}^{t}ds\;h\left(s\right)\right)\right\}\;, \end{array}$$

(25) $$\begin{array}{c} h\left(s\right)=-iH+H_{B}\left(s\right)\;. \end{array}$$

The term −iH implements Euclidean evolution (“gradual cooling”), while the time-dependent piece $H_{B}\left(s\right)$ implements a Brownian *p*-SYK evolution [73]

(26) HB(s)=ip/2∑1≤i1<...<ip≤NgIp(s) ψi1…ψip ,withgIp(s)gIp(s′)¯=JNNp δ(s−s′) .

The time evolution generated by (26) is the continuous-time analog of a random quantum circuit. As such, it drives the initial state toward typicality in a controlled manner. The gradual cooling stabilizes the energy of the state, allowing it to approach a typical TPS at finite temperature.

The correspondence with the interior geometry, and in particular with its spatial isometry, follows from the fact that the random circuit develops a steady state for large enough *t*. More precisely, upon coarse graining over the Brownian couplings, the average preparation corresponds to Euclidean evolution by a Euclidean time *t*,

(27) $$\bar{W\left(t\right)⊗W{\left(t\right)}^{*}}=e^{-tH_{\mathrm{eff}}}\;,$$

for the time-independent effective Hamiltonian,

(28) Heff=H1+H1¯*−JN2Np∑1≤i1<...<ip≤Nip2ψi11…ψip1−(−i)p2ψi11¯…ψip1¯2 .

This class of Hamiltonians was extensively studied in [74]. Because (28) is gapped with an O(1) gap in the large-*N* limit, the evolution (27) for t≫1 prepares the ground state of (28) (more precisely, since (28) has O(N) first excited states, one needs t≫O(logN) to enter the steady regime), which corresponds to the steady state of the random quantum circuit. For $N^{2(p/q-1)}\ll J/J\ll 1$, the ground state admits a semiclassical description in terms of a spatial wormhole connecting two asymptotic AdS boundaries [74]. (The steady state is ensemble equivalent to the canonical Gibbs state $\rho_{\beta }$ at a coupling-dependent effective inverse temperature β=β(J/J). The microscopic coupling strength J/J is tuned to recover the temperature of the black hole β(J/J)=β in the low-temperature phase of SYK. In particular, this requires J/J≪1. The consequence of this is that caterpillar microstates (23) satisfy the TPS condition in the large-*N* limit.)

The average norm over the ensemble of microstates is precisely captured by the Euclidean evolution (27). Consequently, the dominant saddle point geometry of the GPI evaluating the average norm is a Euclidean version of an eternal traversable wormhole metric [27,28]

(29) $$ds^{2}=\frac{d\rho^{2}+dx^{2}}{\sin^{2}\left(\rho \right)}\;,\;\mathrm{with}\;0\leq x+x_{\infty }≲t\;.$$

At any fixed *x*, this geometry prepares the semiclassical dual to the ground state of $H_{\mathrm{eff}}$. The boundary particle’s trajectory sits at $\rho =\rho_{\partial }\propto \epsilon$ and $\rho =\pi -\rho_{\partial }$, where $\rho_{\partial }$ is fixed by the boundary value of the dilaton. Each constant *x* slice prepares the semiclassical dual to the ground state of (28). The spatial isometry follows from the steadiness of this state in the preparation of the microstates. (Using the Markovian property of the Brownian couplings, $W\left(t\right)=W\left(t-t^{\prime }\right)\;W^{\prime }\left(t^{\prime }\right)$ (with $W^{\prime }\left(t^{\prime }\right)$ an independent realization), one may insert operators at arbitrary points along the path-integral preparation of the microstates. After coarse graining over the random couplings, this implies that correlation functions coincide with those of the steady state whenever $t^{\prime }\gg 1$ and $t-t^{\prime }\gg 1$. It then follows that the corresponding Euclidean geometry must develop a Euclidean time translation invariance.) The Euclidean spacetime is illustrated in Figure 6.

Note that the analytic continuation of (29) relevant for the black hole interior spacetime (22) is not the eternal traversable wormhole (global AdS_2_) section, which would be $x=it_{w}$, but rather the “cosmological” continuation $\rho =\frac{\pi }{2}+it_{c}$. Consequently, even if the matter stress-energy tensor $T_{\mu \nu }$ violates the null energy condition in the eternal traversable wormhole section, it does respect it in the caterpillar interior.

**Randomness and the black hole interior.** Caterpillars are the gravitational avatars of random quantum circuits whose circuit depth is set by *t*. As such, the microstates become quantitatively more and more random as *t* is increased. The precise property is that the ensemble of caterpillar microstates (corresponding to all the different realizations of the Brownian couplings at fixed *t*) forms an approximate quantum state *t*-design of the black hole [27,28,75] (a *t*-design is an ensemble of states whose statistical moments are indistinguishable from random (typical TPSs) up to *t* moments). Thus, quantitatively, caterpillar microstates form generic black hole microstates.

Additionally, the parameter *t* sets the average geometric length of the caterpillar interior geometries. This implies a quantitative relation between a geometric property of the interior, namely its length, to a microscopic property of the states, namely their degree of randomness [27,28], in a way that is reminiscent of “complexity = geometry” proposals [76].

**Higher dimensions and simple models.** In principle, caterpillars can be constructed in AdS/CFT. In this case, one expects to be able to replace the SYK operators driving the Brownian evolution by a collection of relevant operators in the CFT dual to bulk fields that create the irregular matter structure of a caterpillar. However, this has not yet been made precise. In [27], an ansatz for the coarse-grained geometry was given in terms of the analytic continuation of a Euclidean eternal traversable wormhole geometry, assuming a perfect fluid stress-energy tensor. To have a complete construction, one would need to consistently find the metric and the stress tensor from the large number of bulk fields with suitable “double trace” boundary conditions. In bottom–up models of AdS_3_ gravity with matter, this was accomplished in [77].

Relatedly, the multi-shell states of Section 2.2 serve as simple toy models for spherically symmetric caterpillars in higher dimensions. In these models, each shell operator is drawn independently from an ensemble (e.g., shells of different masses or types of particles). The ensemble of states created in this way has semi-quantitative similarities with caterpillar states [27].

### 2.5. Topological States

All of the constructions discussed above involve a non-vanishing bulk stress-energy tensor in the black hole interior. It is therefore natural to ask how black hole microstates arise in pure gravity. A particularly interesting setting is pure gravity in three dimensions with negative cosmological constant, where the gravitational field equations admit locally AdS_3_ solutions of the form [30,31,32]

(30) $$ds^{2}=-dt^{2}+\cos^{2}\left(t\right)\;d\Sigma_{g,n}^{2}\;,$$

where $d\Sigma_{g,n}^{2}$ is the constant negative curvature metric on the Riemann surface $\Sigma_{g,n}$ of genus *g* and *n* boundaries. Such surfaces are uniformized in the hyperbolic disk $\Sigma =H^{2}/\Gamma$ via a discrete subgroup Γ of PSL(2,R) isometries. More explicitly, $d\Sigma_{g,n}^{2}=e^{2\omega }dzd\bar{z}$ and the conformal factor ω satisfies the Liouville equation $\partial_{z}\partial_{\bar{z}}\;\omega =\;e^{2\omega }/4$, with suitable boundary conditions imposed at the boundary of the fundamental domain of Γ.

**Figure 7:**
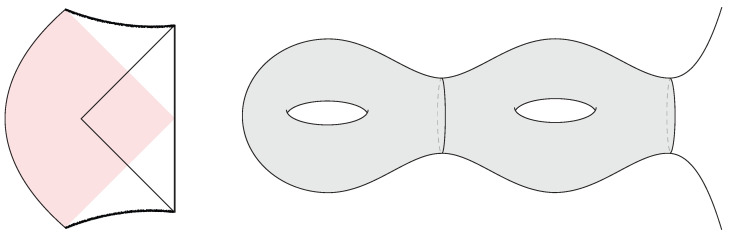
A genus 2 microstate of the three-dimensional black hole. On the left, a section of spacetime (not a Penrose diagram) where we restrict to an interval submanifold of $\Sigma_{2,1}$. The metric (30) covers the red subregion. On the right, the surface $\Sigma_{2,1}$ (where the gray part is $\Sigma_{2,1}\left(L\right)$).

Topological interior states of the three-dimensional black hole are constructed by choosing a spatial slice $\Sigma_{g,1}$ of genus *g* and a single boundary [33]. In this case, $\Sigma_{g,1}=\Sigma_{g,1}\left(L\right)\cup \tilde{\Sigma }\left(L\right)$ is decomposed into a trumpet geometry $\tilde{\Sigma }\left(L\right)$ of fixed geodesic length $L\equiv 2\pi r_{h}$, where $r_{h}$ is the horizon radius, along with a bordered Riemann surface $\Sigma_{g,1}\left(L\right)$ of genus *g* and a geodesic boundary of length *L*. The latter has 6g−4 real moduli, and varying these (within the moduli space of $\Sigma_{g,1}\left(L\right)$) produces different black hole interior states. One can also consider interior states with different genera by varying *g*. For all such states, the metric (30) describes a spacetime subregion, including part of the interior, as represented in Figure 7.

**Microstates.** The microstates of the putative holographic CFT_2_ on a spatial circle are formally defined as

(31) $$|\Psi \rangle =\frac{e^{-\frac{\beta }{4}H}|\Sigma_{g,1}\rangle }{\sqrt{\langle \Sigma_{g,1}|e^{-\frac{\beta }{2}H}|\Sigma_{g,1}\rangle }}\;.$$

Here, $\beta =4\pi^{2}/L$, and $|\Sigma_{g,1}\rangle$ denotes the state prepared by a Euclidean CFT path integral on a Riemann surface $\Sigma_{g,1}\left(2\pi \right)$ of genus *g* with a single geodesic boundary of unit radius.

The correspondence with the interior geometry follows from the Euclidean continuation $t=it_{E}$ of (30), which yields the Euclidean metric

(32) $$ds^{2}=dt_{E}^{2}+\cosh^{2}\left(t_{E}\right),d\Sigma_{g,1}^{2}\;.$$

The Euclidean asymptotic boundary is located at $t_{E}=\pm \infty$. The two parts of the asymptotic boundary are connected through $\partial \Sigma_{g,1}$. The saddle (32) contributes to the norm of the microstate $e^{-\frac{\beta }{4}H}|\Sigma_{g,1}\rangle$. The microstate (31) is defined in the conformal frame in which the hyperbolic trumpet is mapped to a flat cylinder of length β/4, characteristic of plumbing constructions. The geometry (32) is expected to provide the dominant semiclassical saddle for general interior moduli, provided that β is sufficiently small, for which the wavefunction (31) is dominated by the dense spectrum.

**Multi-particle states.** Interior states with massive particles admit explicit constructions in three dimensions [34,35,36]. These can be viewed as generalizations of the dust shell states described in Section 2.2 to configurations without spherical symmetry. In this setting, one considers extensions of the metric (30) to Riemann surfaces $\Sigma_{g,k,1}$ containing *k* punctures, whose corresponding uniformizing subgroups Γ include elliptic elements of PSL(2,R). In particular, this allows for spatial slices with g=0. The topological states described above can be related to the multi-particle states by analytically continuing the conformal dimensions of some of the conical defects beyond the black hole threshold [34,35,36].

**Other generalizations.** Other constructions based on quotients AdS_3_$/\Gamma^{\prime }$, with $\Gamma^{\prime }$ being a discrete subgroup of the full isometry group of AdS_3_, yield topological microstates of the rotating three-dimensional black hole. These interiors are generally not time-symmetric.

### 2.6. Other Proposals

**Figure 8:**
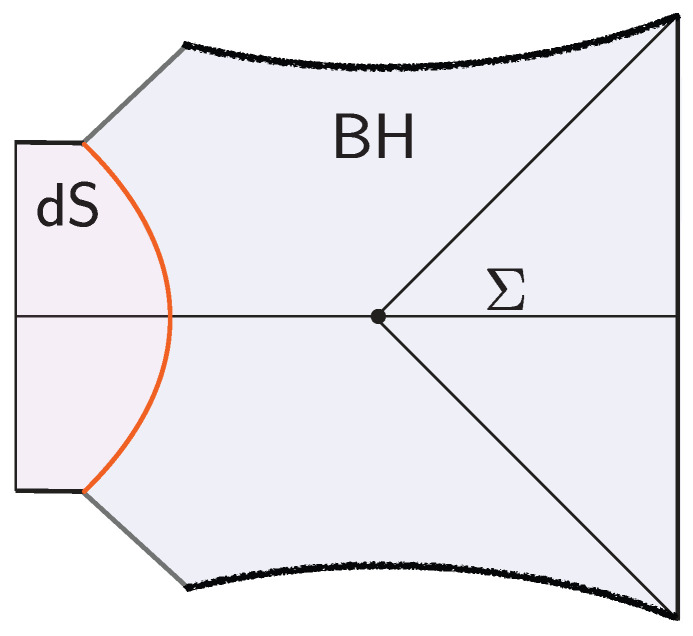
Lorentzian spacetimes for a de Sitter interior state. The gray slices connecting the black hole singularity to the asymptotic dS boundary depends on specific evolution and may be singular.

**de Sitter interior states.** Interface branes can also be used to model processes in which two distinct vacua (metastable and stable) coexist in the thin wall approximation. An example is the Coleman–de Luccia decay of the false (metastable) vacuum into the true (stable) vacuum mediated by an instanton. Another case is the creation of metastable states above the barrier with sufficiently high energy. If the metastable vacuum has a positive energy density, the bubble of the false vacuum inflates. However, for this to be possible for long enough times, the inflating bubble must lie behind the black hole horizon [78]. In [37], black hole interior states with de Sitter inflating bubbles were constructed, which were connected to the Schwarzschild–AdS solution through a pure tension brane. The Penrose diagram of such interiors is shown in Figure 8.

The microscopic interpretation of this construction remains an open question. One reason is that in the Euclidean preparation, the brane’s worldvolume does not reach the Euclidean asymptotic boundary [38]. As a result, the brane cannot be straightforwardly identified with a local (in Euclidean time) operator insertion or with a boundary condition in a putative Euclidean CFT preparation of a pure state. Additionally, the resulting spacetime includes (parts of) de Sitter asymptotic boundaries. These issues obstruct a simple interpretation of these geometries as CFT microstates. (Relatedly, two distinct versions of the holographic entanglement entropy prescription, referred to as “orthodox” and “heterodox” in [79], can be defined based on different choices of the homology constraint. These point to different CFT interpretations of the de Sitter interior state either as a pure state or as a mixed state.)

**Figure 9:**
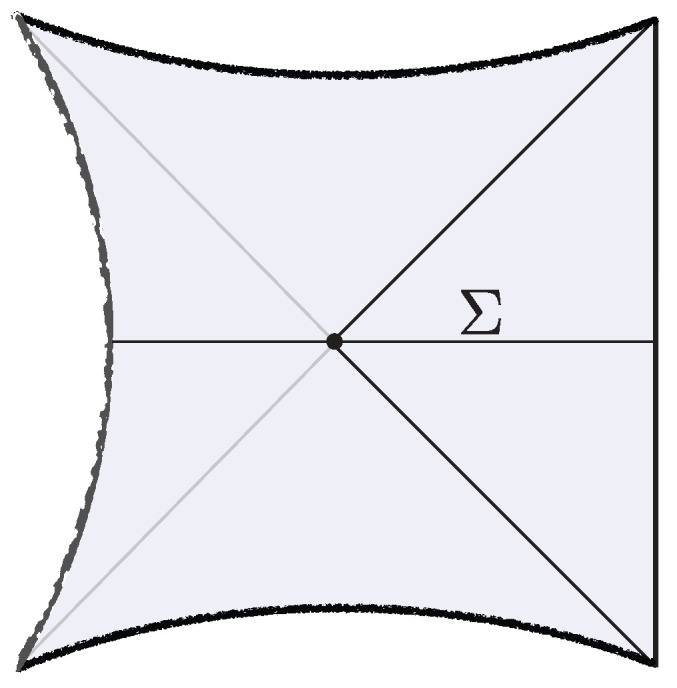
Proposal for the interior geometry dual to a typical microcanonical black hole microstate. The semiclassical spacetime terminates at the left radial boundary.

**Typical microcanonical black hole microstates.** Another proposal, somewhat different in spirit from the constructions discussed above, is that typical microcanonical pure states correspond to eternal black hole interiors extending a finite radial distance behind the horizon [39,40]. This proposal is based on the “mirror operator algebra” construction of [80] (see [81] for aspects of the Tomita–Takesaki theory). The basic assumption is that typical microcanonical states are TPSs even as measured by sufficiently low-frequency modes of the bulk radiation (corresponding to super-scrambling timescales). As such, they should admit thermal correlations with suitable analyticity properties in Euclidean time, which allows for the definition of a “mirror operator” purifying algebra that is analogous to the left CFT algebra of generalized free fields in the thermofield double state. This suggests that the geometry is like an eternal black hole beyond the horizon, as represented in Figure 9.

The underlying assumptions and the physical meaning of the mirror algebra, however, remain somewhat unclear [82,83]. Regarding the assumptions, it is not obvious that any of the microstates discussed above satisfy the required conditions, since their interior geometries can differ significantly from that of the eternal black hole. A common feature of the constructions above is the presence of matter in the interior, so that infalling observers jumping into the black hole a few scrambling times in the past would typically encounter high-energy collisions. At the same time, this does not necessarily imply that such interior matter is detectable by simple operators (“precursors”) at sufficiently early times. Since the matter is not injected through a precursor, detecting it may instead require more complex, non-local probes that are sensitive to the specific microstate. Moreover, the explicit construction of Section 2.4 indicates that interior microstates approximate typical TPSs whose interiors become populated with matter rather than resembling the eternal black hole. Finally, the physical interpretation of the radial boundary in the proposal [39,40] has yet to be fully clarified.

## 3. Entangled Microstates of Multiple Black Holes

The Hilbert space of *n* black holes contains product microstates $|\Psi \rangle =|\Psi_{1}\rangle ⊗\ldots ⊗|\Psi_{n}\rangle$ that semiclassically consist of *n* disconnected black hole interior states, such as those discussed in the previous section. Here, the notion of a microstate, or TPS, refers to a product of thermal states

(33) $$|\Psi \rangle \;∼\;\rho_{\beta }⊗\ldots ⊗\rho_{\beta },$$

assuming all black holes have the same inverse temperature β. Tensoring overcomplete families for each black hole generates an overcomplete family in the product Hilbert space.

In addition to product states, the interiors can appear spatially connected. This is expected to be the case provided the underlying microstates are highly entangled [84,85,86]. We now review several constructions of entangled microstates with connected interiors. The microstates can again be expressed in TPS form (2), where $H=\sum_{i=1}^{n}H_{i}$ is, in this case, a sum of Hamiltonians for each black hole $H_{i}$ that performs the cooling, and |*W*〉 is, in general, an entangled state.

### 3.1. Einstein–Rosen Bridges

For two black holes (n=2), a particularly well-studied entangled microstate is the thermofield double (TFD) state of two holographic CFTs [87]

(34) $$|\sqrt{\rho_{\beta }}\;\rangle =\;\frac{1}{\sqrt{Z(\beta )}}\sum_{i}e^{-\frac{\beta }{2}E_{i}}\;|E_{i}\rangle_{L}⊗{|E_{i}^{*}\rangle }_{R}\;,$$

where $|E_{i}^{*}\rangle =\Theta E_{i}$ for an antiunitary operator Θ (which for CFTs is canonically chosen to be CRT). We write $|\sqrt{\rho_{\beta }}\rangle$ for the two-sided state corresponding to the operator $\sqrt{\rho_{\beta }}$ via the operator–state isomorphism induced by the antiunitary map. We can write the TFD in TPS form as $|\sqrt{\rho_{\beta }}\rangle \;\propto \;\exp\left(-\frac{\beta }{4}H\right)|I\rangle$ for $H=H_{L}+H_{R}$ and |*I*〉 the infinite-temperature TFD state.

**Figure 10:**
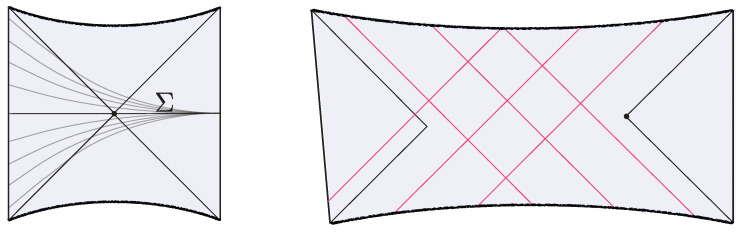
Unperturbed TFD evolution (**left**) and six-shockwave geomery (**right**).

The semiclassical dual to the TFD is the eternal black hole with the quantum fields in the Hartle–Hawking state [87], as illustrated in Figure 10. The correspondence follows from the GPI evaluation of the partition function Z(β), which is interpreted as the norm of the TFD state prepared by the Euclidean CFT path integral on a cylinder of length β/2. For temperatures 1/β above the Hawking–Page transition, the dominant bulk saddle is the Euclidean Schwarzschild–AdS black hole. The reflection-symmetric slice of this geometry Σ prepares the semiclassical Hartle–Hawking state describing two black holes connected by a spatial wormhole, which is known as an Einstein–Rosen (ER) bridge.

Since the TFD is the canonical purification of $\rho_{\beta }$, it is clearly a TPS according to our definition. Moreover, one can consider more general microstates of the form

(35) $$|\Psi_{t}\rangle =e^{-itH_{L}}|\sqrt{\rho_{\beta }}\;\rangle \;,$$

where *t* parametrizes Lorentzian time evolution generated by the left CFT Hamiltonian. Evolving the state in time corresponds, on the bulk side, to selecting a different spacelike slice of the same eternal black hole geometry, anchored at a later boundary time, as shown in Figure 10. In the gray bulk slicing, which can be defined in terms of maximal volume slices, the ER bridge grows with time, eventually entering a regime of linear growth. Importantly, these ER bridges are highly non-generic, as all of them provide vacuum initial data.

The time-evolved TFD states (35) are not overcomplete in the full Hilbert space of the two black holes, since they are restricted to the diagonal energy subspace annihilated by $H_{L}-H_{R}$. However, restricted to this subspace, they form an overcomplete family of microstates within the relevant microcanonical window.

**Shockwave geometries.** In [53,54], generalizations of the TFD time evolution were explored by introducing perturbations in the form of “precursor” operators $W\left(t\right)=e^{-itH_{L}}O_{L}e^{itH_{L}}$. Here, O is a spherical dust shell operator of some small energy *E* close to the boundary at a time *t* defined by the Heisenberg evolution. By interchanging past and future insertion times, it is possible to build a multi-shockwave geometry shown in Figure 10. The corresponding microstates take the form

(36) $$|\Psi_{t_{1},\ldots ,t_{K}}\rangle \;\propto \;W\left(t_{K}\right)\ldots W\left(t_{2}\right)W\left(t_{1}\right)|\sqrt{\rho_{\beta }}\;\rangle \;.$$

In the shockwave geometry, the relevant backreaction parameter is the boosted energy of the shock, α=E/Mexp(2πt/β), where *M* is the ADM energy of the original black hole. This parameter becomes large if the shocks are thrown at super-scrambling times $t\gg t_{*}=\beta \log\left(E/M\right)$.

The microstates $|\Psi_{t_{1},\ldots ,t_{K}}\rangle$ are more generic than the TFD in that they do not lie within the diagonal subspace, and their energy wavefunctions will be erratic if $t_{i}\gg t_{*}$. Nevertheless, they are not expected to form an overcomplete family in the microcanonical Hilbert space of the two black holes, since they arise from gravitational collapse. In particular, consistency requires K≪M/E, so that the total energy injected by the shocks does not significantly increase the mass of the left black hole.

**Two-sided interior states.** While the shockwave geometries discussed above involve matter injected from the boundary, the Euclidean preparation allows matter (or other features) to be placed directly in the interior. This leads to two-sided versions of the dust shell, caterpillar, and topological microstates reviewed in Section 2. For dust shell states, the microstates are known as partially entangled thermal states (PETS) [9,10,11,12,13,15,16] and take the form

(37) $$\left|\Psi \rangle =\right|e^{-\tau H}\;Oe^{-\tau H}\rangle \;.$$

where O denotes the dust shell operator; in AdS_2_, O corresponds to a massive field operator. (In SYK, O may be taken as an O(N)-length Majorana string, or a sum thereof, expected to behave as a PSL(2,R) primary in the infrared. Extending the conformal primary description to such “heavy” operators is non-trivial, however, since they backreact in the large-*N* saddle [88]. Appendix A discusses a closely related higher-dimensional CFT analog for dust shell operators.) For small values of τ, the corresponding semiclassical interior is described by a two-sided black hole containing a single shell (or, in AdS_2_, a particle). As reviewed in Section 2.2, this description follows from evaluating the Euclidean preparation using the GPI while incorporating the backreaction of the shell through the junction conditions (or, in AdS_2_, via the backreaction of the particle on the Schwarzian boundary). (The EoW particle interior states reviewed in Section 2.1 can be formed as $Z_{2}$-quotients of two-sided dust particle states. Alternatively, the single-sided dust shell interior states of Section 2.2 are obtained by making the Euclidean evolution asymmetric, $|\;e^{-\tau_{L}H}\;O\;e^{-\tau_{R}H}\rangle$, and then taking $\tau_{L}\to \infty$, so that the left system decouples (leaving an additional AdS spacetime associated with the ground state of the left CFT).)

**Figure 11:**
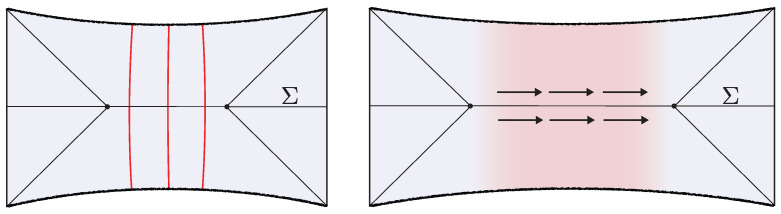
Three dust shell ER bridge (**left**) and ER caterpillar (**right**). In the caterpillar geometry, the black hole interior develops an approximate spatial isometry (represented by the black arrows).

More generally, multi-particle or multi-shell PETSs are described by microstates of the form

(38) $$|\Psi \rangle =|e^{-\tau_{1}H}O_{1}e^{-\tau_{2}H}O_{2}\ldots e^{-\tau_{n}H}O_{n}e^{-\tau_{n+1}H}\rangle ,$$

as illustrated in Figure 11. The corresponding interiors contain ER bridges with multiple shells. The cooling times $\tau_{1},\ldots ,\tau_{n+1}$ are constrained by the ADM energies of the two black holes but are otherwise free parameters that control the interior geometry.

In the same vein, two-sided caterpillar states can be defined as [27,28,29]

(39) $$|\Psi_{t}\rangle =|e^{-\frac{\beta }{4}H}\;W\left(t\right)e^{-\frac{\beta }{4}H}\rangle \;,$$

where W(t) is the finite-temperature random circuit defined in (24). The interior geometry shown in Figure 11 contains an approximate homogeneous ER bridge of O(t) length, supported by a matter distribution, which below the inhomogenity scale develops an irregular structure.

Lastly, topological ER bridges in three-dimensional pure gravity are described by (30) with spatial slices given by a Riemann surface $\Sigma_{g,2}$ of genus *g* and two boundaries. The surface $\Sigma_{g,2}$ admits a decomposition into a bordered Riemann surface $\Sigma_{g,2}\left(L,L\right)$ of genus *g* with two geodesic boundaries of length $L=2\pi r_{h}$ together with two trumpet geometries $\tilde{\Sigma }\left(L\right)$. These geometries correspond to microstates of the form

(40) $$|\Psi \rangle =\frac{e^{-\frac{\beta }{4}H}|\Sigma_{g,2}\rangle }{\sqrt{\langle \Sigma_{g,2}|e^{-\frac{\beta }{2}H}|\Sigma_{g,2}\rangle }}\;,$$

where $H=H_{L}+H_{R}$ is the sum of the CFT Hamiltonians, and $|\Sigma_{g,2}\rangle$ is the state prepared by the Euclidean CFT path integral on the Riemann surface $\Sigma_{g,2}\left(2\pi ,2\pi \right)$ of genus *g* with two geodesic boundaries of unit radius. The correspondence requires β to be sufficiently small so that the GPI evaluation of the norm is dominated by the connected phase. For the g=0 case, $\Sigma_{0,2}\left(2\pi ,2\pi \right)$ is infinitely thin and prepares the infinite-temperature TFD, $|\Sigma_{0,2}\rangle =|I\rangle$, so that the corresponding microstate (40) reduces to the TFD.

**BPS black holes.** For BPS black holes described by N=2 super JT gravity [89,90] (such as $\frac{1}{16}$-BPS black holes in AdS_5_$\times S^{5}$), one can construct PETS in the BPS sector [11,12,15]

(41) $$|\Psi_{\mathrm{BPS}}^{Q}\rangle =|e^{-\infty H}O_{1}e^{-\infty H}O_{2}\ldots e^{-\infty H}O_{n}e^{-\infty H}\rangle \;,$$

where *H* is the Hamiltonian in the fixed *R*-charge sector of charge *Q*, which is quantized in units of the fundamental charge $1/\hat{q}$. Even if the operators $O_{i}$ are not BPS, the infinite Euclidean evolution exp(−∞H) projects them into the BPS sector.

These microstates correspond to the intrinsically quantum mechanical states of two entangled BPS black holes. They are not semiclassical geometries. Instead, their structure involves large quantum fluctuations of the throat. This is reflected in the wavefunction of the empty wormhole, which is described as the ground state of super Liouville quantum mechanics for the geodesic length [11,12].

### 3.2. Multiple Black Holes

**Figure 12:**
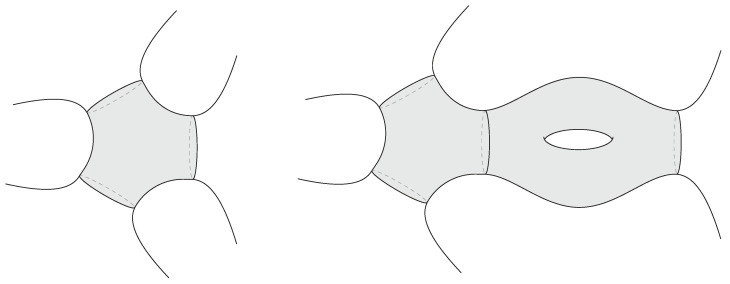
Fully connected interior genus-0 (**left**) and genus-1 (**right**) microstates of n=3 three-dimensional black holes, where the gray region is $\Sigma_{0,3}\left(L,L,L\right)$ and $\Sigma_{1,3}\left(L,L,L\right)$, respectively.

Multi-party entangled states of more than two black holes (n>2) can yield fully connected black hole interiors. These have been explicitly constructed in three-dimensional pure gravity in [30,31,32,91], as they are solutions of the form (30) for a Riemann surface $\Sigma_{g,n}$ of genus *g* with more than two boundaries n>2. (See also [79,92] for explicit multi-boundary wormhole initial data in four dimensional flat space pure gravity.) In this case, the surface $\Sigma_{g,n}$ admits a decomposition into a bordered Riemann surface $\Sigma_{g,n}\left(L,\ldots ,L\right)$ of genus *g* with *n* geodesic boundaries of length *L* together with *n* trumpet geometries $\tilde{\Sigma }\left(L\right)$, as represented in Figure 12. As explained in Section 2.5, the corresponding microstates have the form

(42) $$|\Psi \rangle =\frac{e^{-\frac{\beta }{4}H}|\Sigma_{g,n}\rangle }{\sqrt{\langle \Sigma_{g,n}|e^{-\frac{\beta }{2}H}|\Sigma_{g,n}\rangle }}\;,$$

where $H=\sum_{i=1}^{n}H_{i}$ is the sum of CFT Hamiltonians and $|\Sigma_{g,n}\rangle$ is the CFT state prepared by the Euclidean path integral on the Riemann surface $\Sigma_{g,n}\left(2\pi ,\ldots ,2\pi \right)$ of genus *g* with *n* geodesic boundaries of unit radius. When β is much smaller than all other moduli, the completely connected interior is expected to dominate, although the phase diagram as a function of the Riemann surface moduli is complicated.

The geometric connectivity of the interior constrains the allowed forms of multipartite entanglement of the corresponding black hole microstates, which are required to satisfy the holographic entropy inequalities [93]. As an example, GHZ states with four or more parties are incompatible with smooth interior geometries, as they violate the monogamy of mutual information [91,94].

## 4. Semiclassical State Counting of Black Hole Entropy

The constructions reviewed so far highlight a familiar tension between bag of gold interiors and black hole entropy. Making the microstates explicit sharpens the associated paradox in which interior states appear to overcount the Bekenstein–Hawking entropy (1). In this section, we review the setups in which this tension is resolved by carefully counting states using the GPI [8,14,15,16,29]. In these cases, Euclidean spacetime wormholes control the overlap moments between interior states, rendering the would-be orthogonal microstates non-orthogonal. Accounting for this non-orthogonality yields a finite entropy in agreement with the black hole entropy (1).

The GPI state counting derivations of black hole entropy that we review are closely connected to earlier applications of the GPI while at the same time extending them to a broader class of interior states. In particular, they are structurally parallel to the GPI derivation of the Page curve [8] (see also [10,36] in higher dimensions), which was framed in a different context [95,96,97], but where replica wormholes capture nontrivial overlap moments between semiclassical interior states, leading to a reduction of the coarse-grained entropy of the black hole and, consequently, to the entropy of the Hawking radiation after the Page time. Related ideas, including the appearance of “null states” in low-dimensional “third quantized” gravitational systems, have been discussed in [98]. Related wormhole-induced effects were discussed in [99,100].

### 4.1. State Counting with the GPI

Given a family of Ω black hole microstates

(43) $$F_{\Omega }=\left\{|\Psi_{1}\rangle ,\ldots ,|\Psi_{\Omega }\rangle \right\}\;,$$

the notion of quantum statistical entropy is defined in relation to the Hilbert space dimension spanned by this family

(44) $$\begin{array}{c} d_{\Omega }=\dim\left(\mathrm{span}\left\{F_{\Omega }\right\}\right)\;. \end{array}$$

The dimension is encoded in the rank of the Ω×Ω Gram matrix of overlaps of the family

(45) $$\begin{array}{c} G_{ij}=\langle \Psi_{i}|\Psi_{j}\rangle \;\left(i,j=1,\ldots ,\Omega \right)\;, \end{array}$$

(46) $$\begin{array}{c} d_{\Omega }=\mathrm{rank}\left(G\right)\;. \end{array}$$

The rank can be evaluated in ways other than direct diagonalization, which are suitable for semiclassical methods, such as the one we review below. One particularly convenient method is to analytically continue the moments of the Gram matrix and evaluate its rank by taking the limit

(47) $$d_{\Omega }=\lim_{n\to 0}\;\mathrm{Tr}\left(G^{n}\right)\;.$$

Each microstate $|\Psi_{i}\rangle$ is prepared by a Euclidean path integral in the underlying CFT or SYK model. The overlap $G_{ij}$ is given by a Euclidean path integral on a closed contour, while its moments $G_{ij}^{n}$ are computed from Euclidean path integrals on replicated copies of the system. In gravity, these moments can be evaluated using the GPI in the large-*N* expansion of the holographic system. We denote gravitational overlaps computed in this way with an overline.

(48) $$\bar{G_{ij}},\;\bar{G_{ij}^{2}},\;\ldots ,\;\bar{G_{ij}^{n}}\;.$$

We represent the gravitational spacetime contributions diagrammatically as(49)
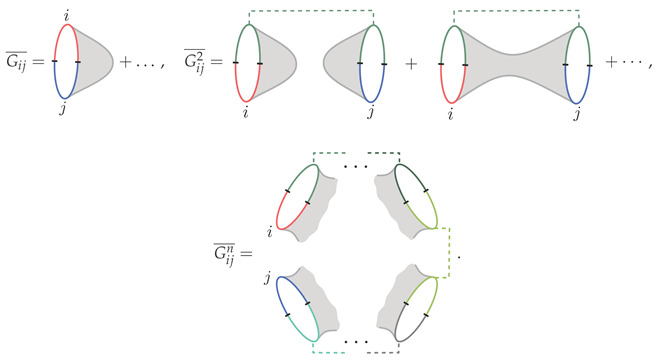


For pictorial purposes, we draw overlaps between two-sided states, where each colored half-boundary contour corresponds to the Euclidean time in the preparation of a microstate in two copies of the holographic system. Each microstate is labeled by an abstract index. Dashed lines denote sums over indices. In the final diagram for $\bar{G_{ij}^{n}}$, we leave implicit the GPI evaluation of all allowed topologies (connected, partially connected, or disconnected) compatible with the presence of *n* boundaries. (The Euclidean path integral prepares unnormalized states, so the GPI computes unnormalized overlaps, which are subsequently normalized by the GPI evaluation of the norms of the states.)

**Bag of gold paradox and its resolution.** The bag of gold paradox arises from interpreting $\bar{G_{ij}}$ as the genuine Gram matrix of microstate overlaps. In the absence of fine tuning, the rank of this matrix simply equals the number of states. (Determining $\mathrm{rank}(\bar{G})$ explicitly in concrete examples can be technically challenging, although (50) holds in the absence of fine tuning. In simple toy models where bulk states carry flavor indices, this is straightforward, since the overlap at the disk level is $\bar{G_{ij}}=\delta_{ij}$ [8]. For families of dust shell interior states, one can similarly achieve $\bar{G_{ij}}=\delta_{ij}$ either by taking the limit $|m_{i}-m_{j}|\to \infty$ or by considering sufficiently heavy shells built from distinct particle species [13]. A noteworthy case where this is under more microscopic control is [29], where one sees that interior caterpillars orthogonalize at the disk level if the wormhole lengths are of the order of the scrambling time of the black hole. In other situations, obtaining an orthogonal family at the disk level requires diagonalizing $\bar{G_{ij}}$.)

(50) $$\mathrm{rank}(\bar{G})=\Omega \;,$$

which grows without bound as the number of states Ω increases. In particular, when $\Omega >e^{S_{\mathrm{BH}}}$, the resulting Hilbert space dimension would exceed that allowed by the black hole entropy, leading to a bag of gold paradox. All the interior families reviewed in the previous sections, except for EoW brane interiors, are overcomplete in this sense.

The resolution to the bag of gold paradox comes from the fact that one must instead consider

(51) $$\bar{d_{\Omega }}=\bar{\mathrm{rank}(G)}=\lim_{n\to 0}\;\mathrm{Tr}\left(\bar{G^{n}}\right)\;,$$

as the true dimension spanned by the family of microstates. In particular, connected contributions captured by wormholes dominate the moments and yield the saturation of the dimension,

(52) $$\begin{array}{c} \bar{d_{\Omega }}=\min\left\{\Omega ,e^{S_{\mathrm{BH}}}\right\}\;, \end{array}$$

precisely at the right value, which gives a quantum statistical derivation of black hole entropy (1) as

(53) $$\log\bar{d_{\infty }}=S_{\mathrm{BH}}\;.$$

**Figure 13:**
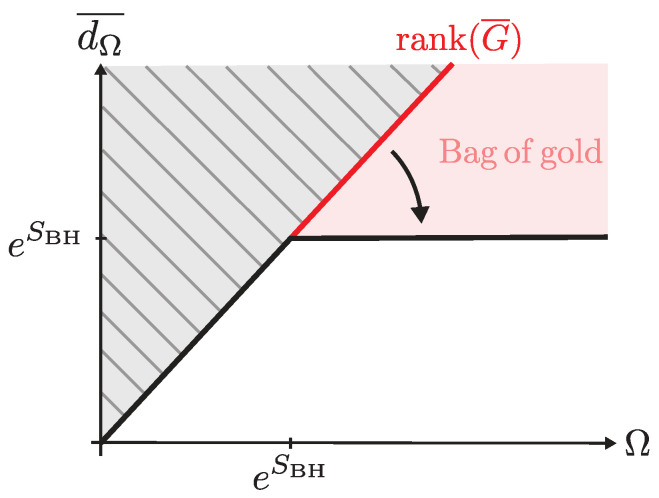
State counting using the GPI. In the absence of fine tuning, the rank of the Gram matrix initially is the number of states Ω. When $\Omega >e^{S_{\mathrm{BH}}}$, the rank of the coarse-grained overlap matrix $\bar{G}$ continues this linear growth (red curve), signaling a bag of gold regime. Non-perturbative wormhole corrections account for non-orthogonalities in the microscopic Gram matrix, whose cumulative effect produces linear dependence among states of the family, and the corresponding plateau for its rank (black curve). The saturation value of the log dimension yields the Bekenstein–Hawking entropy.

We illustrate this situation in Figure 13.

A few comments are in order. First, the saturation value of the dimension can be evaluated particularly simply in the limit Ω→∞ when the GPI overlaps are symmetric under permutations of the indices, which are conditions that are often satisfied. In this case, the moments of the Gram matrix simplify to

(54) Tr(Gn)¯∼∑i1≠…≠in=1Ω〈Ψi1|Ψi2〉〈Ψi2|Ψi3〉⋯〈Ψin|Ψi1〉¯=ΩnZnZ1n ,

where $Z_{n}$ is the cyclic overlap between unnormalized states computed by the GPI,

(55) $$Z_{n}\equiv \bar{\langle \Psi_{1}|\Psi_{2}\rangle \langle \Psi_{2}|\Psi_{3}\rangle \cdots \langle \Psi_{n}|\Psi_{1}\rangle }\;.$$

In Section 4.2, we review the fully connected replica-symmetric wormholes that control the evaluation of $Z_{n}$ in the explicit cases of interest. In this case, the saturation value of the dimension can be directly derived from the analytic continuation

(56) $$\bar{d_{\infty }}=\lim_{n\to 0}Z_{n}\;.$$

Secondly, the microstates reviewed in Section 2 and Section 3 live in much larger Hilbert spaces—either infinite-dimensional holographic CFTs or $2^{N/2}$-dimensional SYK models. As a result, Equations (52) or (53) do not directly apply to them. To perform a proper black hole entropy counting, one must instead restrict to a finite-energy microcanonical window of the holographic system, which is centered at energy E=M corresponding to the black hole mass and of width ΔE∼1/β. The precise value of the width, as long as it is O(1/β), is unimportant to reproduce the large-*N* entropy.

Given this, one must translate the full spacetime wormhole result for the overlaps into the overlap moments of the microcanonical Gram matrix

(57) $$Z_{n}^{E}\equiv \bar{\langle \Psi_{1}|\Pi_{E}|\Psi_{2}\rangle \langle \Psi_{2}|\Pi_{E}|\Psi_{3}\rangle \cdots \langle \Psi_{n}|\Pi_{E}|\Psi_{1}\rangle }\;,$$

where $\Pi_{E}$ denotes the orthogonal projector into the microcanonical window. This step is direct because the wormholes we review fix the energies of each asymptotic boundary to be the same. The microcanonical projection simply restricts that common energy to the chosen window and picks out the corresponding contribution from the full amplitude, which is an energy integral

(58) $$\begin{array}{c} Z_{n}=\int dE\varrho \left(E\right)\;G{\left(E\right)}^{n}\;, \end{array}$$

(59) $$\begin{array}{c} \Rightarrow \;Z_{n}^{E}=\left(\Delta E\right)\varrho \left(E\right)\;G{\left(E\right)}^{n}\;. \end{array}$$

Here, ϱ(E) and G(E) are functions arising from the GPI contribution of the wormhole. In all relevant cases for this review, one finds $\varrho \left(E\right)=e^{S_{\mathrm{BH}}\left(E\right)}/\Delta E$. (The microcanonical overlaps $Z_{n}^{E}$ can also be evaluated directly using the GPI and appropriate boundary conditions for the energy projectors [101]. The result (59) arises from “pac-man” versions of the replica-symmetric wormhole [16].)

The resulting microcanonical dimension spanned by the family is then

(60) $$\bar{d_{\infty }\left(E\right)}=\lim_{n\to 0}Z_{n}^{E}=e^{S_{\mathrm{BH}}\left(E\right)}\;.$$

in agreement with the black hole entropy as the quantum-statistical entropy.

A final remark concerns the case of multiple black holes. When the GPI state counting is performed using states such as those reviewed in Section 3, the resulting log dimension obtained from the GPI is simply the sum of the individual black hole entropies. This additivity follows from the contribution of the corresponding replica wormholes to the overlap moments, whose structure mirrors that of (58). In the case of two black holes, the contributions are of the form

(61) $$\begin{array}{c} Z_{n}=\int dE_{1}dE_{2}\;\varrho \left(E_{1}\right)\;\varrho \left(E_{2}\right)G{\left(E_{1},E_{2}\right)}^{n}\;, \end{array}$$

(62) $$\begin{array}{c} \Rightarrow \;Z_{n}^{E}={\left(\Delta E\right)}^{2}\varrho {\left(E\right)}^{2}\;G{\left(E,E\right)}^{n}\;. \end{array}$$

**Microscopic interpretation.** The overlines denote quantities computed using the GPI, but they admit a microscopic interpretation as disorder averages in the dual holographic system. This viewpoint has emerged from studies of spacetime wormholes in holography (see [102,103,104,105] and references therein). In the cases relevant for this review, the overlap moments can be understood as arising from an average over the erratic matrix elements of “heavy” operators associated with the matter supporting the black hole interior states [34,50,106,107,108,109], as these operators enter explicitly in the wavefunctions of the microstates. It is important to emphasize that this averaging is not fundamental; rather, it provides an effective description of the microscopic overlap moments within the scope of the GPI. This approximation is sufficient for extracting the dimension of the Hilbert space spanned by a given family of states, as the Hilbert space dimension is a coarse quantity that is self-averaging over disorder realizations.

The quantity $\bar{d_{\Omega }}$ in (51) should therefore be viewed as a “quenched average” dimension, which retains information of rank(G) for the individual microscopic instance of the Gram matrix. On the other hand, $\mathrm{rank}(\bar{G})$ by itself carries no direct information about the Hilbert space in which the microstates reside beyond providing an upper bound on its dimension.

**Typical TPSs.** In some limits, the GPI treats the microstates as independent Gaussian random vectors [8,13,14,15,16]. Correspondingly, $G_{ij}$ is effectively an instance of an ensemble of Wishart random matrices. In the large dimension limit, the average spectral density of Wishart matrices follows the so-called Marchenko–Pastur distribution. This can be derived directly using the GPI from its moments through the so-called resolvent matrix

(63) $$R_{ij}\left(\lambda \right)\;\equiv \;{\left(\frac{1}{\lambda 1-G}\right)}_{ij}\;=\;\frac{1}{\lambda }\;\delta_{ij}+\sum_{n=1}^{\infty }\;\frac{1}{\lambda^{n+1}}\;{\left(G^{n}\right)}_{ij}\;.$$

A standard result in linear algebra is that the eigenvalue density D(λ) of $G_{ij}$ is governed by the discontinuity of the trace of the resolvent along the imaginary axis,

(64) $$D\left(\lambda \right)=\lim_{\epsilon \to 0}\frac{1}{2\pi i}\left(R(\lambda -i\epsilon )-R(\lambda +i\epsilon )\right)\;.$$

where we denote the trace as $R\left(\lambda \right)=\sum_{i=1}^{\Omega }R_{ii}\left(\lambda \right)$.

The GPI evaluation of the resolvent matrix obeys a Schwinger–Dyson equation, which is manifest in the Gaussian wormhole diagrammatics [8,13,14,15,16]. The mircocanonical Schwinger–Dyson equation can be resummed and solved explicitly, and using (64), one can derive the spectral density $D_{E}\left(\lambda \right)$ of the microcanonical Gram matrix of overlaps. The resulting eigenvalue density is the Marchenko–Pastur distribution [8]

(65) $$\begin{array}{cc} \bar{D_{E}\left(\lambda \right)} & =\frac{e^{S_{\mathrm{BH}}\left(E\right)}}{2\pi \lambda }\sqrt{\;\left[\lambda -{\left(1-\sqrt{\Omega e^{-S_{\mathrm{BH}}\left(E\right)}}\right)}^{2}\;\right]\left[\;{\left(1+\sqrt{\Omega e^{-S_{\mathrm{BH}}\left(E\right)}}\right)}^{2}-\lambda \right]}\\ \; & +\delta \left(\lambda \right)\left(\Omega -e^{S_{\mathrm{BH}}\left(E\right)}\right)\theta \left(\Omega -e^{S_{\mathrm{BH}}\left(E\right)}\right)\;, \end{array}$$

where again $e^{S_{\mathrm{BH}}\left(E\right)}$ arises from the replica wormhole contribution to the moment $Z_{n}$.

From (65), the finite-Ω formula (52) follows explicitly: when $\Omega \leq e^{S_{\mathrm{BH}}\left(E\right)}$, the Gram matrix has no zero eigenvalues and its rank is maximal, while when $\Omega >e^{S_{\mathrm{BH}}\left(E\right)}$, the Gram matrix develops $\Omega -e^{S_{\mathrm{BH}}\left(E\right)}$ zero eigenvalues and its rank saturates at precisely $e^{S_{\mathrm{BH}}\left(E\right)}$.

### 4.2. Replica Wormholes and Entropy

We now review the replica wormhole contributions to the overlap moments that are responsible for the saturation value of the Hilbert space dimension in specific cases.

**Figure 14:**
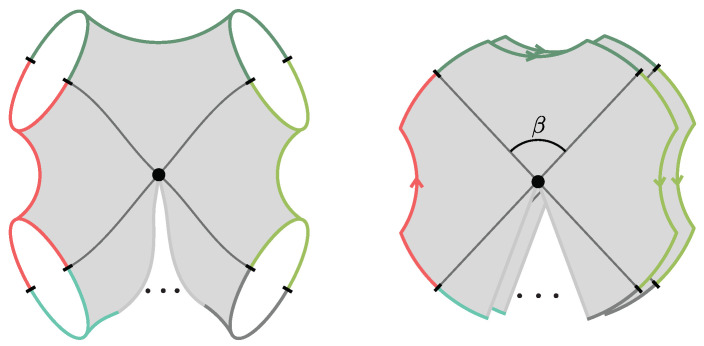
(**Left**) general pinwheel wormhole controlling the cyclic overlap moments. The manifold branches around the minimal surface (black dot). (**Right**) when the matter forming the microstates is sufficiently heavy, it lies in the Euclidean asymptotic boundary, and the wormhole reduces to (two copies of) the Euclidean black hole. The total Euclidean periodicity of the geometry is nβ.

**EoW particle states.** In [8], one models the EoW particle states of Section 2.1 as carrying a semiclassical “flavor” index *i* corresponding to the precise boundary condition imposed by $|P_{i}\rangle$. The disk level overlap then gives $\bar{G_{ij}}=\delta_{ij}$, and this yields a bag of gold paradox, since there, Ω can be as large as $2^{N/2}$. At the level of the GPI, the statistics from the wormhole diagrammatics is Gaussian random. (We do not expect the KM microstates to behave as typical TPSs for sufficiently high moments. Reproducing such statistics may require acting on each individual state $|P_{i}\rangle$ with an independent random unitary.) This wormhole is dubbed the “pinwheel wormhole” illustrated in Figure 14. Its contribution can be evaluated using the technique introduced in [110] where one divides the geometry into Hartle–Hawking states and hyperbolic polygons using the length basis of JT gravity. This yields the general expression (58) for the two functions

(66) $$\begin{array}{c} \varrho \left(E\right)=\frac{e^{S_{0}}}{2\pi^{2}}\sinh\left(2\pi \sqrt{2\phi_{b}E}\right)\;, \end{array}$$

(67) $$\begin{array}{c} G\left(E\right)=e^{-\beta E}\;2^{1-2m}\;\Gamma \left(m-\frac{1}{2}+i\sqrt{2\phi_{b}E}\right)\;. \end{array}$$

where $\phi_{b}$ is the boundary value of the dilaton JT. Using (60), the state counting predicts a quantum statistical entropy given by the black hole entropy, which within the semiclassical regime is

(68) $$\log\bar{d_{\infty }\left(E\right)}=S_{0}+2\pi \sqrt{2\phi_{b}E}=S_{\mathrm{BH}}\left(E\right)\;,$$

upon the suitable identification of $S_{0}$ and $\phi_{b}$ with the parameters of the higher-dimensional near-extremal black hole (“extremal entropy” and specific heat, respectively).

**Dust shell states.** For dust shell interior states, the relevant replica wormhole contributing to $Z_{n}$ is a higher-dimensional generalization of the pinwheel wormhole [10,13,14,50]. (See also [36] for the analogous pinwheel wormhole for multi-particle interior states in three dimensions.) As illustrated in Figure 14, this wormhole is constructed by gluing two copies of the Euclidean black hole solution along the worldvolumes of *n* dust shells (for the single-sided dust shell states of Section 2.2, this reduces to a single copy). For details of the geometry and the explicit evaluation of the gravitational action, we refer the reader to [13]. A remarkable simplification occurs in the heavy-shell limit $m_{i}\to \infty$, where the shell worldvolumes localize to the asymptotic region and the solution reduces to the Euclidean black hole geometry, up to shell worldvolume factors that cancel against the normalization $Z_{1}^{n}$. (If, additionally, we take $|m_{i}-m_{j}|\to \infty$, the GPI effectively treats the dust shell states as independent Gaussian random vectors, and one recovers $\delta_{ij}$ for disk level overlaps.) In this case, the contribution of the wormhole is

(69) $$\frac{Z_{n}}{Z_{1}^{n}}={\left(\frac{Z(n\beta )}{Z{\left(\beta \right)}^{n}}\right)}^{\alpha }\;,$$

where α=1,2 depending on whether the states are one or two-sided, respectively. These overlap moments are universal in that they arise across different constructions (single shell, multi-shells, …) in the limit in which the matter supporting the black hole interior is sufficiently heavy. They signal that the corresponding microstates exhibit the same overlap moments as the typical TPSs.

In this regime, the wormhole action has the general form (58) (or (61) for α=2) for

(70) $$\begin{array}{c} \varrho \left(E\right)=\int_{\gamma }\frac{d\beta }{2\pi i}e^{\beta E}Z\left(\beta \right)\;\equiv \rho_{\mathrm{GH}}\left(E\right), \end{array}$$

(71) $$\begin{array}{c} G\left(E\right)=e^{-\beta E}f\left(m\right)\;, \end{array}$$

where γ is a vertical contour in the half-plane with Re β=0+. For α=2, in this case, we have $G\left(E,E\right)=G{\left(E\right)}^{2}$, and f(m) is an energy-independent contribution of a single shell (see [13] for details). In (70), we have used the fact that the result of the integral is the density of states $\rho_{\mathrm{GH}}\left(E\right)∼e^{S_{\mathrm{BH}}\left(E\right)}$ computed by Gibbons and Hawking [4]. Therefore, we find that the GPI state counting of dust shell interior microstates produces a quantum statistical entropy in agreement with black hole entropy

(72) $$\log\bar{d_{\infty }\left(E\right)}=\log\varrho_{\mathrm{GH}}{\left(E\right)}^{\alpha }=\alpha S_{\mathrm{BH}}\left(E\right)\;.$$

**BPS black holes.** For the particle states of BPS black holes described in Section 3.1, the counting is simpler, because one does not need to implement a microcanonical projection, since all of the states lie in the BPS sector. The resulting pinwheel wormhole yields the overlap moment [15]

(73) $$\frac{Z_{n}}{Z_{1}^{n}}={\left(e^{S_{0}}\cos\left(\pi Q\right)\right)}^{2(1-n)}\;,$$

where the term inside the parenthesis corresponds to the zero temperature partition function in the charge *Q* sector, which is evaluated using the GPI [89,90] (the groundstates can only have |Q|<1/2). Using (56), the quantum statistical entropy is

(74) $$\log\bar{d_{\infty }\left(Q\right)}=2S_{0}+2\log\cos\left(\pi Q\right)\;,$$

which agrees with the entropy of the two entangled BPS black holes [89,90].

**Caterpillars.** The GPI state counting of caterpillars is particularly interesting because the parameter *t* labels the family of microstates. For a fixed value of *t*, one chooses Ω distinct microstates, corresponding to caterpillars with different features at sub-inhomogeneity scales but of the same O(t) length. Microscopically, the different states correspond to different realizations of the time-dependent couplings in the preparation of the states. Since Ω is unbounded above, there is a bag of gold paradox for any *t*. The question is whether the GPI state counting derivation of black hole entropy is robust in the sense that it yields the same Hilbert-space dimension for all *t*. The answer was shown to be affirmative in [29] by explicitly constructing the replica wormhole which dominates the overlap moments for $t\gg t_{*}∼\beta \log N$. (The condition $t\gg t_{*}$ arises because distinct caterpillars require preparation times related to the scrambling time to become orthogonal at the disk level, so disconnected contributions are suppressed relative to the fully connected replica wormhole. A separate interesting feature of this setup is that the state counting can be carried out explicitly in microscopic models, since the disorder average induced by the GPI is better understood. At the level of the on-shell wormholes considered here, this disorder average maps to a microscopic average over the Brownian couplings used in the state preparation [29]. This makes it possible to reproduce analogous large-*N* “semiclassical” state countings in systems without a gravity dual, such as infinite-temperature SYK.)

**Figure 15:**
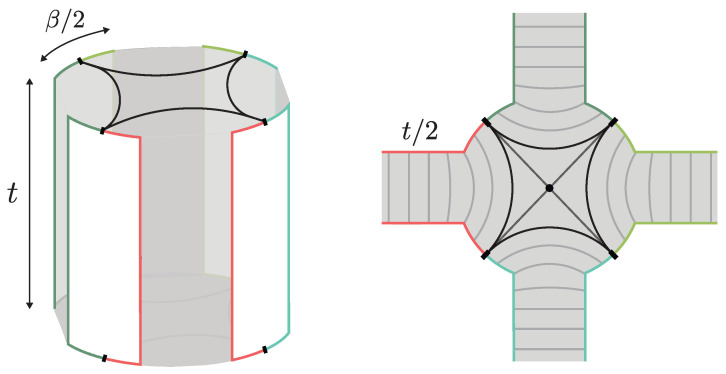
(**Left**) Structure of the birdcage wormhole where the bars of the cage have length *t*. (**Right**) The wormhole is constructed by gluing a global AdS_2_ strip of length *t* to the disk.

The relevant replica wormhole for $Z_{n}$ is the “birdcage” wormhole of Figure 15, namely a two-sided pinwheel geometry in which the two copies are connected by *n* boundary strips of length *t*. It can be also explicitly evaluated in JT by dividing it into Hartle–Hawking preparations, strips, and hyperbolic polygons, and it yields the general expression (61) for the two functions,

(75) $$\begin{array}{c} \varrho \left(E\right)=\frac{e^{S_{0}}}{2\pi^{2}}\sinh\left(2\pi \sqrt{2\phi_{b}E}\right)\;, \end{array}$$

(76) $$\begin{array}{c} G\left(E,E^{\prime },t\right)=\int d\ell \int d\ell^{\prime }\;K_{2i\sqrt{2\phi_{b}E}}\left(4e^{-\ell /2}\right)\;G_{t}\left(\ell ,\ell^{\prime }\right)\;K_{2i\sqrt{2\phi_{b}E}}\left(4e^{-\ell^{\prime }/2}\right), \end{array}$$

where $G_{t}\left(\ell ,\ell^{\prime }\right)$ is the heat kernel of the *ℓ*-particle in an effective potential that can be found in [27,29]. Using (60), the replica wormhole yields the dimension

(77) $$\log\bar{d_{\infty }\left(E,t\right)}=2S_{0}+4\pi \sqrt{2\phi_{b}E}=2S_{\mathrm{BH}}\left(E\right)\;\left(\forall \;t\gg t_{*}\right)\;,$$

which shows that the GPI state counting derivation of black hole entropy is remarkably robust.

### 4.3. Further Aspects

We conclude by discussing several aspects that follow from these state-counting calculations.

**Quantum corrections and negative modes.** The black hole entropy is corrected at subleading orders in $G_{N}$ and yields the so-called generalized entropy. In principle, perturbative corrections can be captured by the GPI state counting by including the one-loop fluctuations of matter fields in the relevant replica wormhole. For dust shell states in the heavy shell limit, due to the universal answer (69), this will yield an answer consistent with the one-loop corrections to the entropy computed by the Gibbons–Hawking density of states [16]. These corrections can be derived more cleanly by performing the GPI state counting in doubly holographic settings [111].

Relatedly, for flatspace black holes or small AdS black holes, there exists a well-known perturbative normalizable instability in the transverse-traceless graviton sector, localized near the horizon, which is interpreted as signaling the thermodynamic instability of the black hole. This instability introduces factors of i throughout the GPI state counting for these black holes. In [112], this issue was resolved by performing a careful Laplace transform to microcanonical overlaps.

**Statistical variances.** Another possible source of corrections is that the GPI computation of the variance of the dimension,

(78) $$\frac{\bar{d_{\infty }^{2}}-{\bar{d_{\infty }}}^{2}}{{\bar{d_{\infty }}}^{2}}\neq 0\;,$$

may be nonzero even if it is very small. Such a variance would originate from connected contributions to moments of the form $\bar{\mathrm{Tr}\left(G^{n}\right)\;\mathrm{Tr}\left(G^{m}\right)}$ after analytically continuing to n,m→0.

It is interesting to ask where this variance originates from. As we have reviewed, the GPI provides a coarse-grained statistical description of the holographic system. We must therefore treat the microstates effectively as a collection of random vectors. For a collection of random vectors drawn from a smooth probability distribution, an undercomplete set of vectors is linearly independent with unit probability even if these vectors are correlated with one another. From this viewpoint, one might therefore expect the dimension computed with the GPI to exhibit no variance at all. In fact, this can be shown explicitly using the GPI for wormholes stabilized by the “matter sector’’, when the matter operators (either particles or shells) have Gaussian diagrammatics [15]. However, the result is expected to hold more generally.

The caveat is that the black hole entropy is defined microcanonically and therefore depends on the Hamiltonian through the choice of energy window. Once a window is fixed, “edge effects’’ can arise: collections of eigenvalues may fluctuate in or out of the window depending on the disorder realization of the Hamiltonian encoded by the GPI. In particular, off-shell wormholes governing spectral correlations can generate a non-vanishing variance. These effects are expected to be extremely small, but they may become relevant for precision tests within this framework.

In this sense, an advantage of the BPS case is that the variance vanishes because there is a semiclassical gap between the BPS black hole and the first excited states [90]. The GPI computation of the variance (78) gives a vanishing result because in N=2 super JT, the off-shell wormhole corrections vanish [15]. In this case, the GPI respects the BPS condition exactly, so these “edge effects” are absent.

**Counting states in pure gravity.** Even though the previous cases show the robustness of state-counting methods with the GPI, it would be interesting to perform the counting in three-dimensional pure gravity, for the topological microstates reviewed in Section 2.5, in particular in relation to the infinite entropy in the semiclassical quantization [33,113].

**Developing a state-counting formalism.** Ideally, one would like to elevate these examples into a more general framework that systematically captures the overcompleteness of semiclassical families of states. Progress along these lines has been made in [114,115,116], where the state-counting ideas reviewed here are placed on a more systematic footing and developed into a more explicit formalism across a range of settings.

## 5. Conclusions

The GPI state-counting derivation of black hole entropy presented in this review has two notable strengths. It is *universal* in that it applies independently of the type of black hole and does not rely on detailed assumptions about the microscopic completion of gravity beyond the existence of such a completion. It is also *robust*, since whenever the counting has been carried out, the result is insensitive to the particular choice of interior families used in the counting.

While the derivations reproduce the “external” description of the black hole as a quantum system with a genuine quantum statistical entropy given by (1), they naturally leave open a basic interpretational question: what is the meaning of the overcompleteness of interior microstates, and what are its physical implications for the black hole interior?

In light of recent progress in holography and the black hole information paradox [8,95,96,97], the prevailing interpretation is that this overcompleteness reflects a limitation of the exterior Hilbert space description to holographically encode generic black hole interior states. In particular, generic (mixed) bulk states in bag of gold spacetimes carry a von Neumann entropy that parametrically exceeds (1). Such large “code subspaces” cannot be unitarily encoded in the exterior microscopic system; instead, they belong to the entanglement wedge of the system that purifies the black hole interior. Whether this “purifier” is truly necessary for an intrinsic description of the black hole interior, or whether an alternative microscopic formulation exists in its absence, remains an open question in quantum gravity.

## Figures and Tables

**Table 1:** Summary of the constructions of black hole microstates. For AdS_2_, the microstates are defined in the Hilbert space of the SYK model, while for AdS_d+1_ with d≥2, they are defined in the Hilbert space of a holographic CFT_d_.

Sec.	Microstates	AdS Dimension	Overcomplete	Refs.
Section 2.1	EoW particle	2	✓	[7,8]
Section 2.2	Dust shell	≥3	✓	[9,10,11,12,13,14,15,16]
Section 2.3	EoW brane	≥3	✗	[10,17,18,19,20,21,22,23,24,25,26]
Section 2.4	Caterpillar	2	✓	[27,28,29]
Section 2.5	Topological	3	✓	[30,31,32,33,34,35,36]
Section 2.6	Other	≥3	-	[37,38,39,40]

## Data Availability

No new data were created or analyzed in this study.

## Appendix A. Dust Shell Operators

From the perspective of the bulk low-energy effective field theory, a dust shell corresponds to a dilute (relative to the cutoff scale) collection of massive particles that is heavy enough to produce an appreciable gravitational field, much like an ordinary astrophysical body. The dust shell operator O is defined as the creation operator for such a macroscopic object. (Recall, for instance, that a dust shell cannot be formed as the coherent state of a heavy free scalar field, as the latter carries pressure and therefore obeys a different equation of state, behaving more like a tensional brane.) One can formally write it down in terms of single-trace CFT operators of low conformal dimension using the extrapolate dictionary, since O creates the collection of particles in the asymptotic AdS region at a radius $r_{\infty }$. The resulting operator is inherently multi-trace, involving $O(N^{2})$ such single-trace operators [51],

(A1) $$O=\prod_{i=1}^{O(N^{2})}\phi_{i}\left(r_{\infty },\theta_{i}\right)\;,$$

where $\theta_{i}\in S^{d-1}$. It might be convenient to moreover smear the operator in the *s*-wave sector (by integrating over the $\theta_{i}$s) to define a homogeneous surface operator. As the operators $\phi_{i}$ are CFT generalized free fields, one may further replace them by their purely creation parts so that the resulting shell operator carries a definite particle flavor charge in the large-*N* limit.

One may be interested in deriving the bulk dynamics of the dust shell directly from the CFT. The challenge is that the 1/N expansion around a fixed “background’’ state breaks down, since the backreaction of the heavy operator cannot be treated perturbatively. This problem should be viewed as a consistency check of AdS/CFT, as the correct description should, by construction, reproduce the bulk effective field theory of gravity coupled to the dust shell. This direction has been mainly pursued in AdS_3_/CFT_2_ bootstrap under the assumption of identity conformal block dominance in the natural OPE channel of the two-point function of O [51,117].

A conveniently simple CFT model of the dust shell operator, which correctly reproduces the physics of the semiclassical bulk two-point function from the CFT, is to effectively model it as a random operator in the energy basis with a smooth envelope function [50],

(A2) $$\langle E_{i}|O|E_{j}\rangle =e^{N^{2}f\left(\bar{E},\omega \right)}R_{ij}\;,$$

where $\bar{E}=\left(E_{i}+E_{j}\right)/2$, $\omega =E_{i}-E_{j}$, and $f(\bar{E},\omega )$ is some O(1) smooth function determined in [50]. The coefficients $R_{ij}$ are random complex numbers of O(1) magnitude and erratic phases. Such a random matrix description of the heavy operator may be justified by invoking a generalized form of the “eigenstate thermalization hypothesis” (ETH) that applies to heavy operators. (Moreover, the $R_{ij}$ are not strictly Gaussian. For instance, the correlator ${\langle O\left(\tau_{1}\right)O{\left(\tau_{2}\right)}^{†}O\left(\tau_{3}\right)O{\left(\tau_{4}\right)}^{†}\rangle }_{\beta }$ (with $0\leq \tau_{1}<\tau_{2}<\tau_{3}<\tau_{4}<\beta$) has a non-vanishing semiclassical connected contribution associated with shell crossing, which is suppressed in the heavy-shell limit m→∞.) In holographic CFT_2_s, this effective statistical characterization can be made more precise in terms of Liouville line defect operators [109] (see [34] for context). In any case, it is important to keep in mind that a dust shell operator is not fundamentally disordered and that (A2) is just a phenomenological model that reproduces the bulk physics in a very simple way.

The statistical model has the advantage of reproducing the effect of wormhole contributions to the GPI. This is understood as indicating that the GPI coarse-grains over the CFT microscopics rather than defining a fundamental average.
